# Supplementary material for: Quantitative characterization of cell niches in spatially resolved omics data
Source: Nat Genet. 2025 Mar 18;57(4):897–909. doi: 10.1038/s41588-025-02120-6 (PMC11985353; doi:10.1038/s41588-025-02120-6)
Supplement: Supplementary file 1 — Supplementary Figs. 1–35, Table 1, Notes 1–12 and Methods. [file 41588_2025_2120_MOESM1_ESM.pdf]

nature genetics

Supplementary information https://doi.org/10.1038/s41588-025-02120-6

Quantitative characterization of cell niches in spatially resolved omics data

In the format provided by the authors and unedited

Supplementary Figures

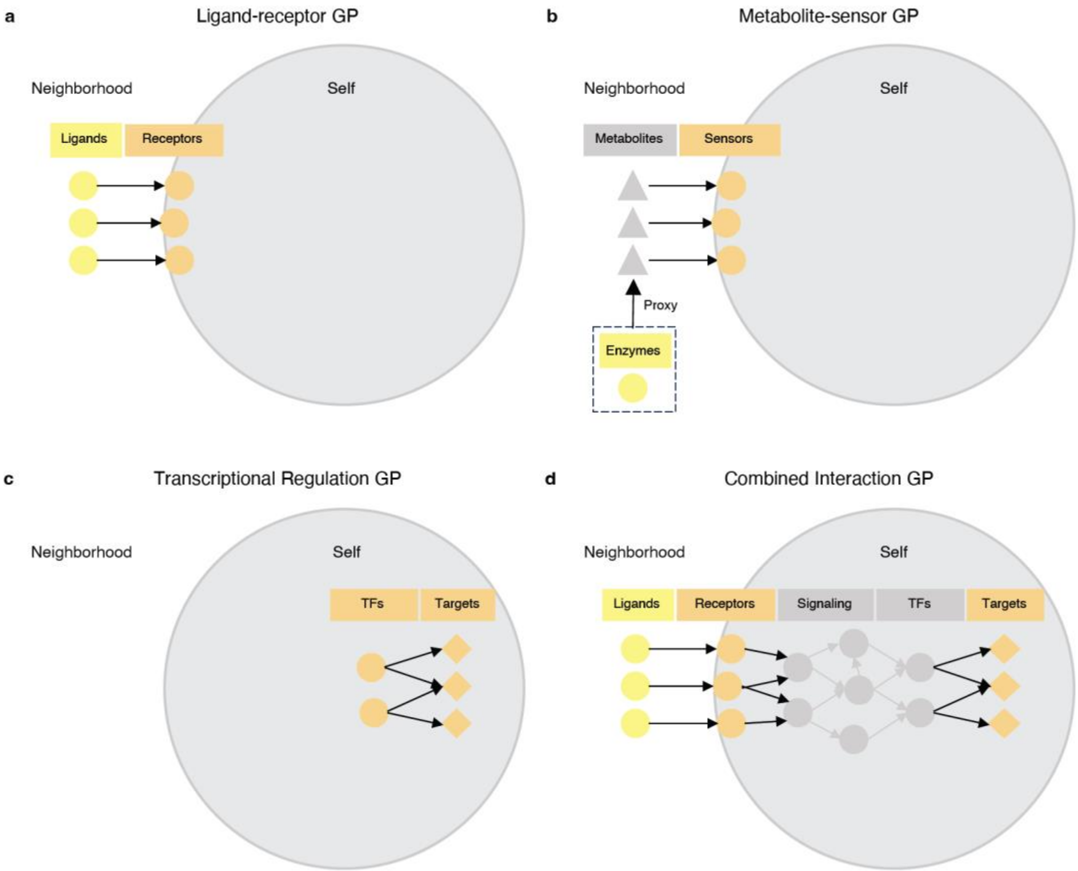

Supplementary Fig. 1 | Categorization of prior programs. a, Ligand-receptor program comprising ligands in the neighborhood component and receptors in the self-component. **b**, Metabolite-sensor program comprising metabolites in the neighborhood component and sensors in the self-component. Enzyme expression serves as a proxy for metabolite presence. **c**, Transcriptional regulation program comprising transcription factors (TFs) and target genes in the self-component. **d**, Combined interaction program comprising ligands in the neighborhood component and receptors and target genes in the self-component. Circles represent proteins, triangles represent metabolites, and rhombi represent genes involved in interactions. For each protein, programs contain the corresponding protein-encoding gene. GP: gene program.

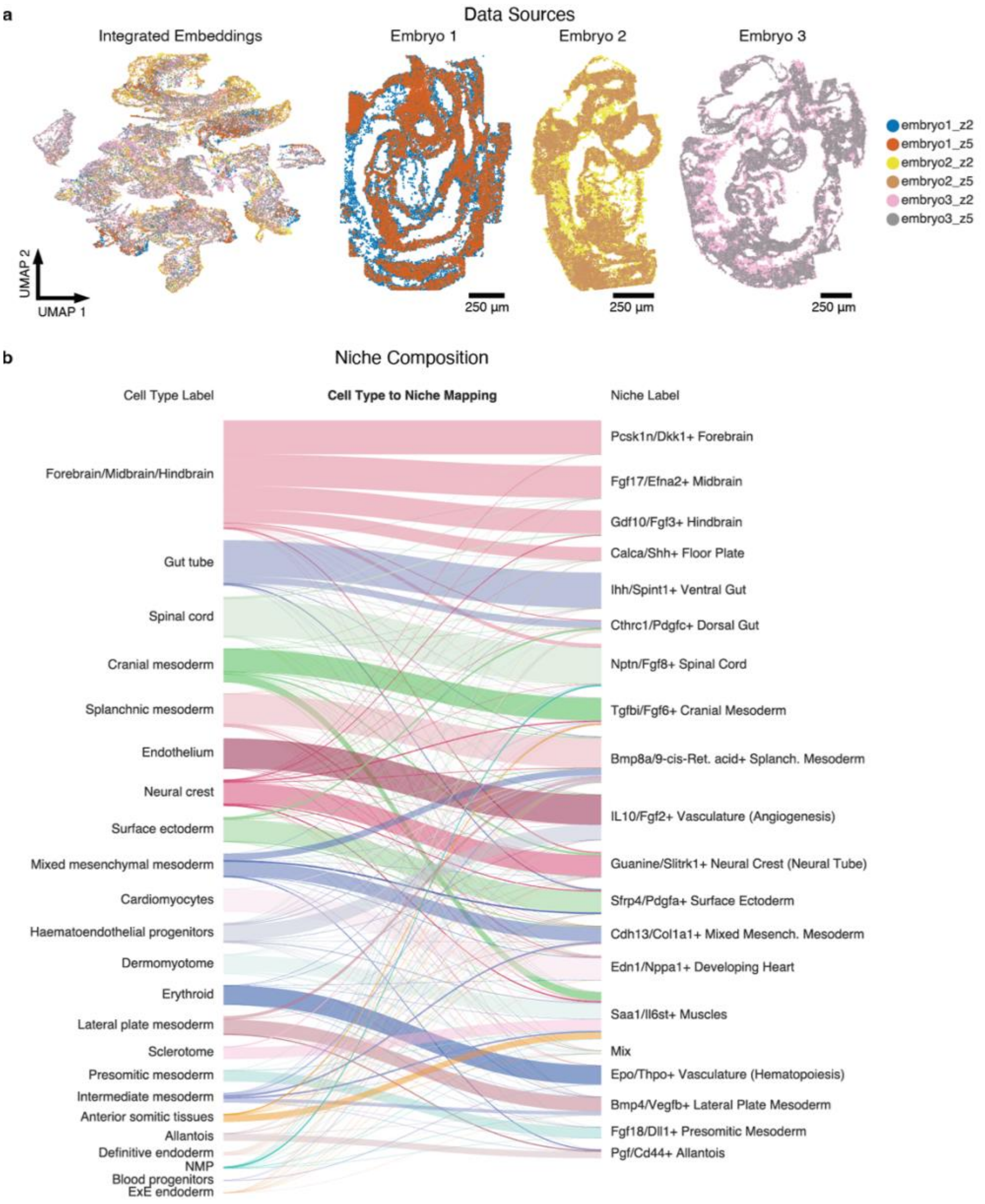

Supplementary Fig. 2 | **Mouse organogenesis tissue section integration and niche composition.** **a**, UMAP of integrated NicheCompass embeddings of three 8-12 somite stage embryo tissues colored by section (data source), which is used as covariate during training to remove batch effects. Next to it the three embryo tissue sections[1]. **b**, Mapping of cell type/region annotations from the original publication to clusters/niches obtained by clustering the NicheCompass embedding space.

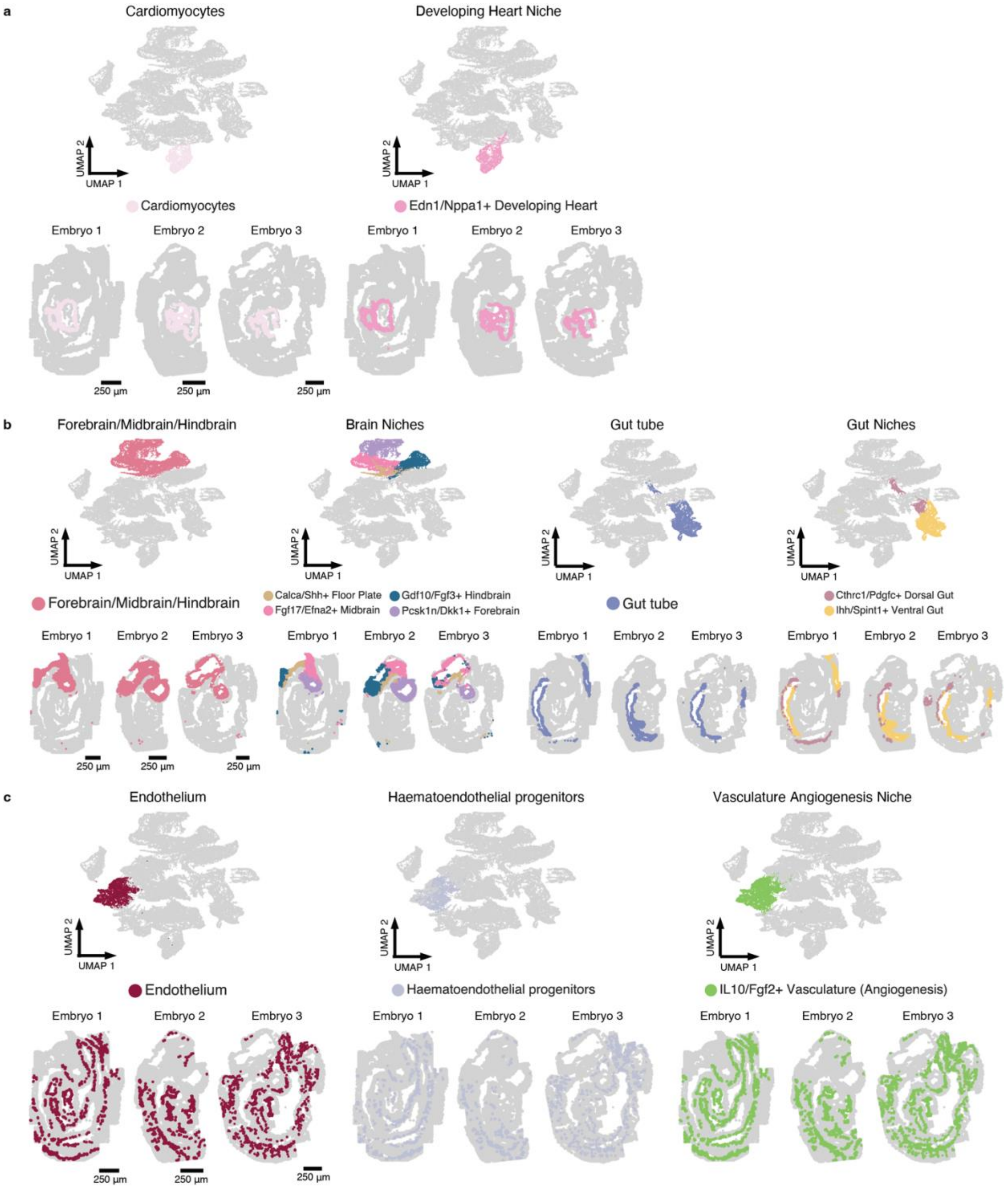

Supplementary Fig. 3 | **Mouse organogenesis niche heterogeneity.** **a**, The developing heart niche as an example of a homogeneous cell population, containing all cells of a single cell type from the original annotations[1], illustrated by an adjacent visualization of cardiomyocyte cells. **b**, Brain and gut niches as examples of homogeneous cell populations with increased resolution compared to the original annotations, illustrated by adjacent visualizations of the forebrain/midbrain/hindbrain and gut tube cells. **c**, The vasculature angiogenesis niche as an example of a niche consisting of heterogeneous cell populations. Only cells belonging to the respective niches are colored.

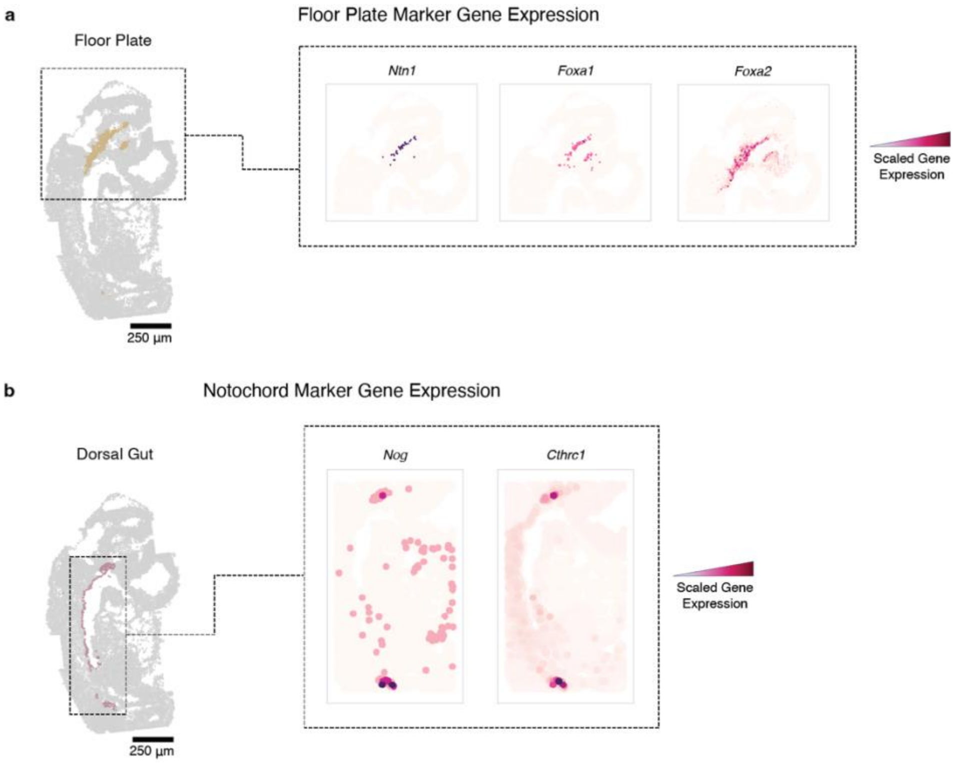

Supplementary Fig. 4 | Niche-specific marker genes. a, The Floor Plate niche in the second embryo, along with the expression of three established floor plate marker genes[2], validating the identification and annotation of the niche. **b**, Expression of two known notochord marker genes[3], validating the localization of this embryonic midline structure within the Dorsal Gut niche.

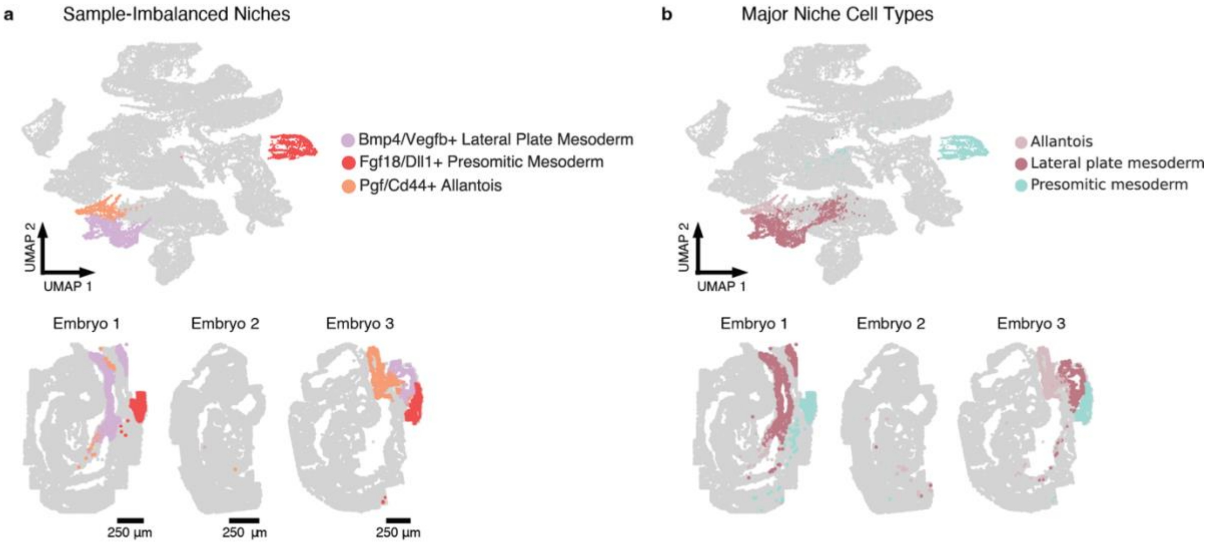

Supplementary Fig. 5 | Imbalanced niches across samples. a, Three niches that were imbalanced across samples, with considerable presence only in the first and third mouse embryo samples. **b**, The three corresponding major cell types constituting the niches from **a** show similar presence in only the first and third mouse embryo samples.

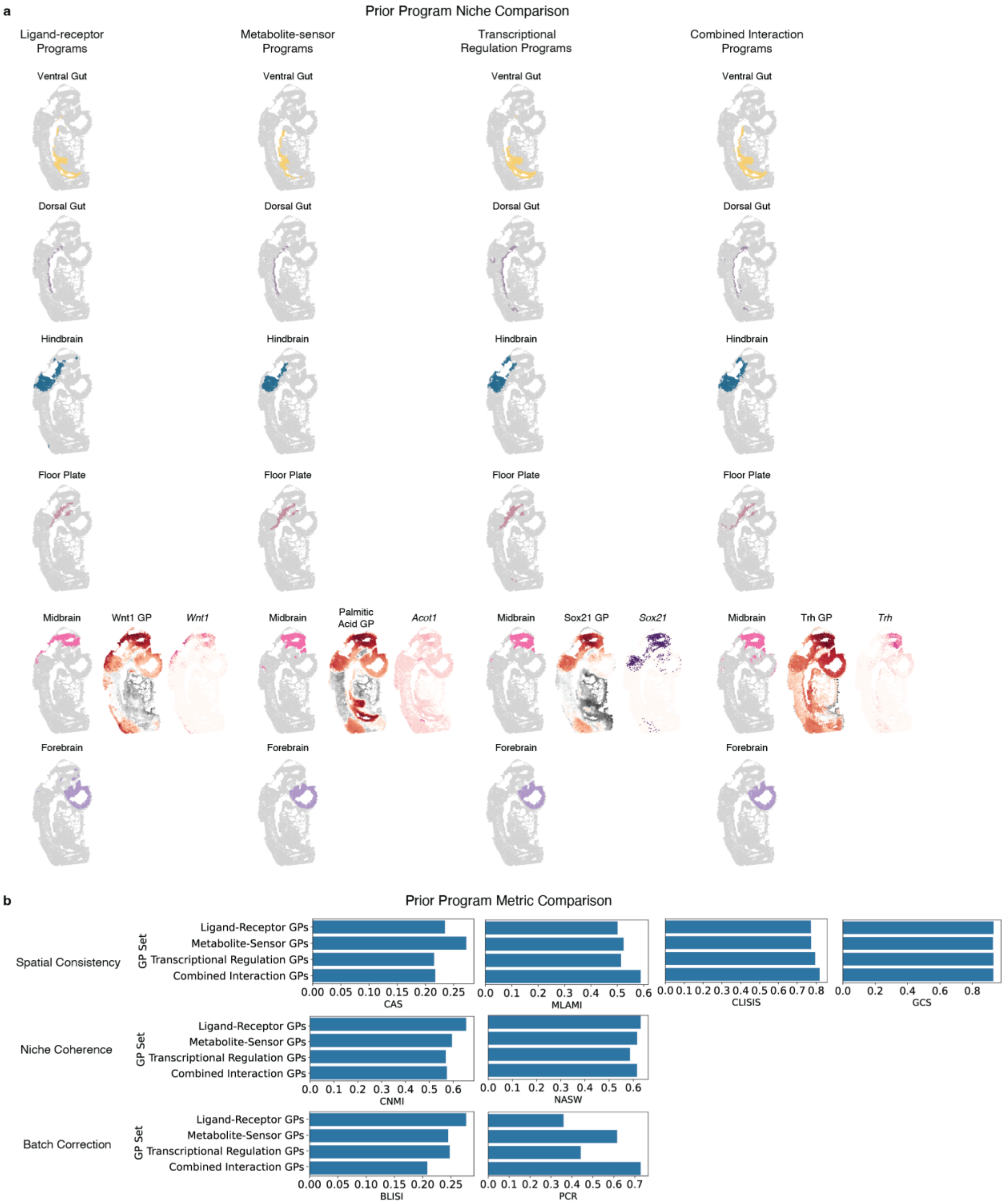

Supplementary Fig. 6 | **Niche and program inference with different program sets. a**, Brain and gut niches identified when only using a specific set of prior programs. All niches from the analysis in Fig. 2 can be recovered independently of the used set of prior programs; however, different prior programs elucidate different aspects of underlying niche biology, highlighted with characterizing programs of the Midbrain niche and their most important genes. **b**, Metrics for the different prior program sets from **a**, showing only slight performance differences. GP: gene program.

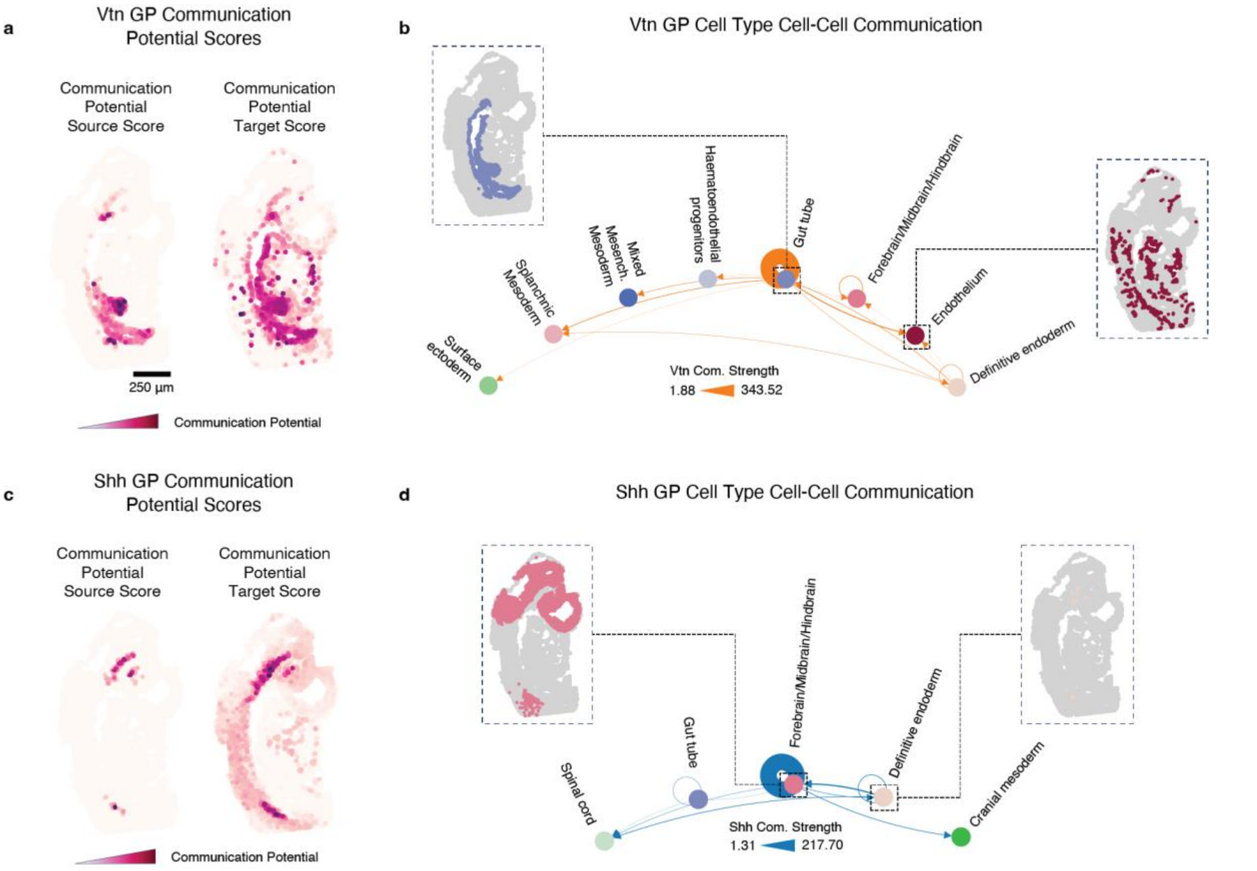

Supplementary Fig. 7 | **Cell-cell communication inference for the Vitronectin and Sonic Hedgehog programs. a**, Source- and target-specific cell-cell communication potential scores for the Vtn combined interaction program. **b**, Cell-pair communication strengths of the Vtn combined interaction program aggregated by cell types, highlighting interaction within gut tube cells and between gut tube and endothelial cells. **c**, Source- and target-specific cell-cell communication potential scores for the Shh combined interaction program. **d**, Cell-pair communication strengths of the Shh combined interaction program aggregated by cell types, highlighting interaction within brain cells and between brain and definitive endoderm cells. GP: gene program. Com. Strength: communication strength.

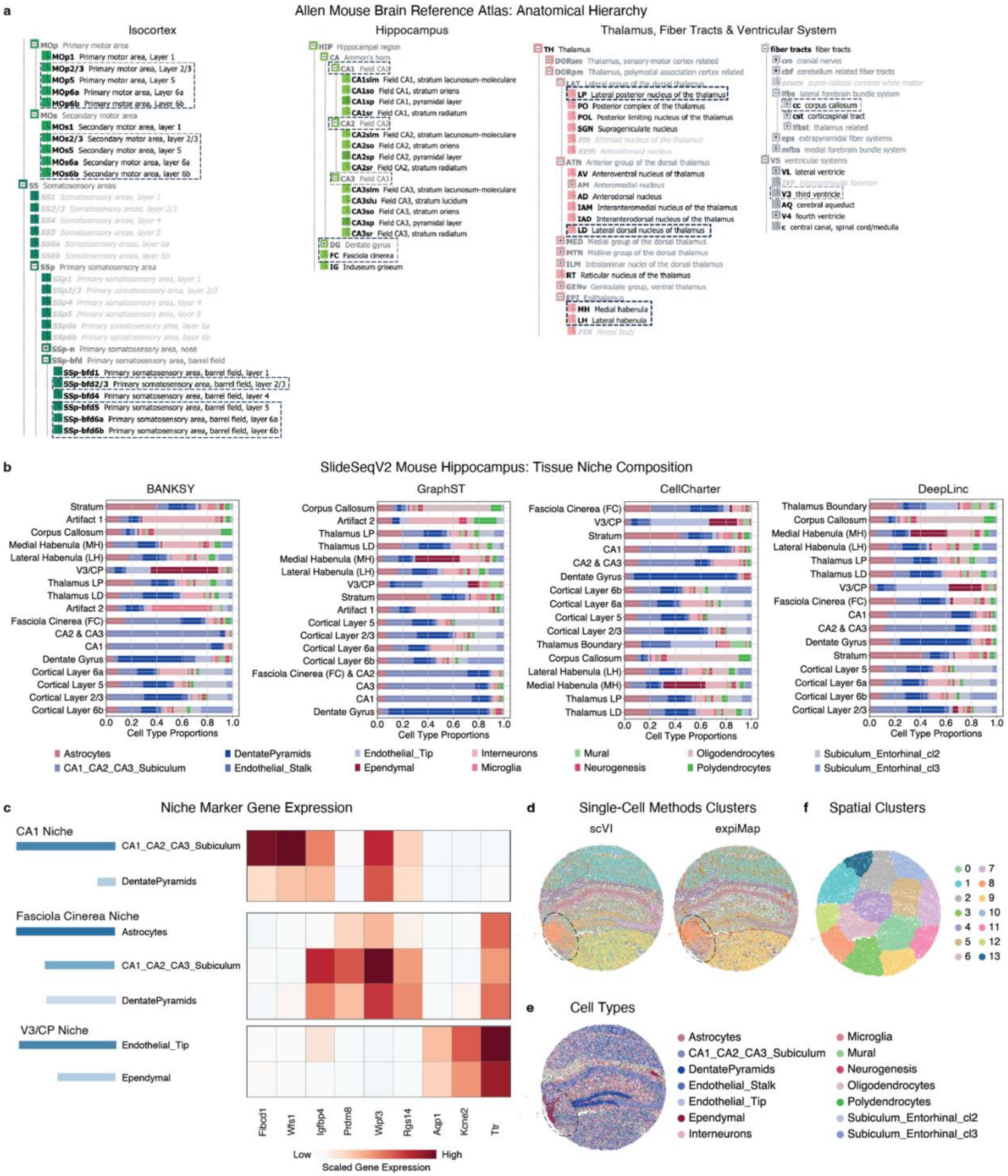

Supplementary Fig. 8 | **Benchmarking on the SlideSeqV2 mouse hippocampus dataset.** **a**, Anatomical hierarchy of the mouse brain from the Allen Brain Atlas[4]. Highlighted are niches identified by NicheCompass. **b**, Cell type composition of niches identified by four similar methods[5-8]. **c**, Expression of marker genes in three adjacent niches[9], delineating niches beyond anatomical location and cell type composition. Each row corresponds to a specific niche. Bar charts on the left display the proportions of the most abundant cell types, with bar color and length indicating relative abundance. Heatmaps display average expression levels of niche-specific marker genes, with color intensity representing mean expression levels. Shown are niche-specific marker genes that are differentially expressed in a niche compared to all other niches. **d**, The mouse hippocampus tissue[10] colored by clusters obtained from two (non-spatial) single-cell methods. Highlighted is a region that shows clusters influenced by spatial effects beyond cell types. **e**, The mouse hippocampus tissue colored by original cell type annotations. **f**, The mouse hippocampus tissue colored by clusters obtained from clustering the spatial coordinates, illustrating spatially uniform, non-biologically meaningful clusters.

a

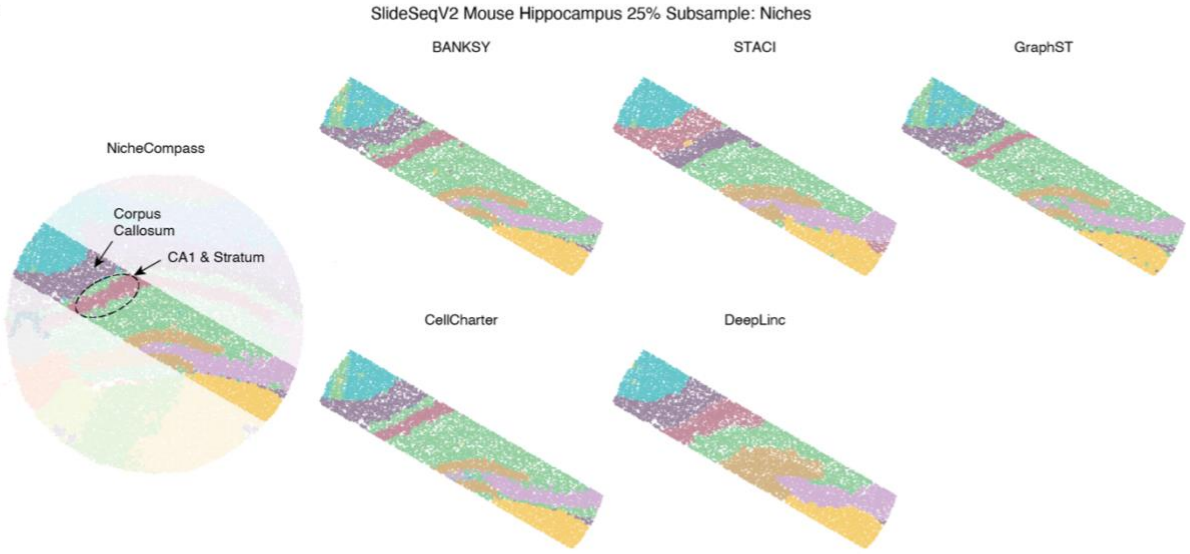

b SlideSeqV2 Mouse Hippocampus 25% Subsample: Metrics

Model	CAS	MLAMI	CLISIS	GCS	NASW	CNMI	Overall Score
NicheCompass	0.850	0.532	0.916	0.873	0.683	0.216	0.678
BANKSY	0.797	0.439	0.909	0.860	0.621	0.243	0.561
STACI	0.821	0.577	0.936	0.921	0.538	0.155	0.418
GraphST	0.642	0.421	0.898	0.910	0.540	0.246	0.410
CellCharter	0.711	0.466	0.883	0.783	0.561	0.239	0.342
DeepLinc	0.574	0.601	0.922	0.799	0.545	0.201	0.327
	Spatial Consistency				Niche Coherence		

Supplementary Fig. 9 | Benchmarking on the SlideSeqV2 mouse hippocampus 25% subsample. a, A 25% subsample of the mouse hippocampus tissue[10], colored by niches identified through clustering of the embedding spaces of NicheCompass and five similar methods[5-8,13]. Cluster numbers and colors are consistent across methods. The tissue annotated with NicheCompass is overlaid with the full dataset results, showing high niche consistency. **b**, Six benchmarking metrics across two categories, spatial consistency and niche coherence, are min-max-normalized and aggregated into an overall score to evaluate the performance of each method on this dataset.

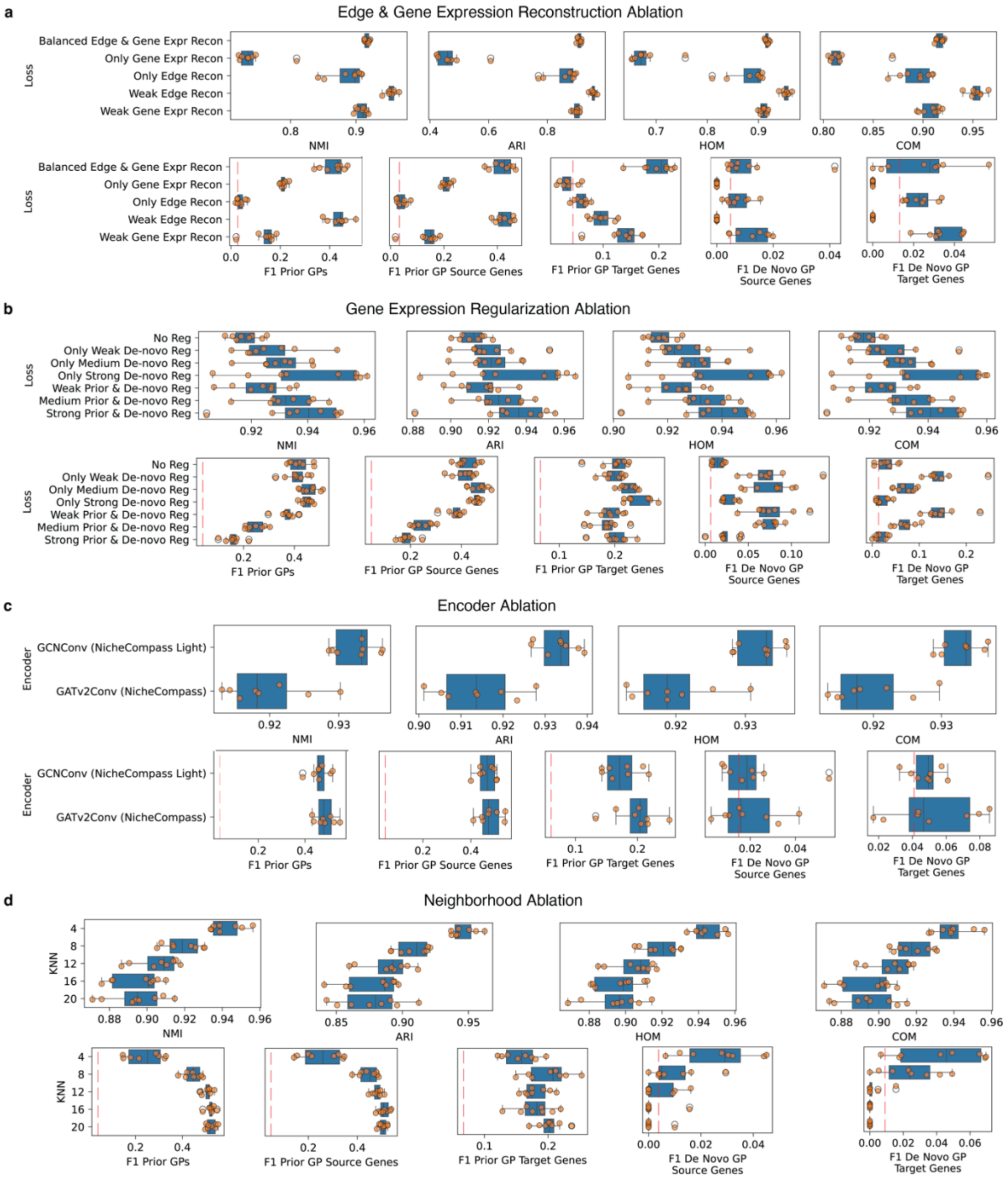

Supplementary Fig. 10 | **Ablation study on simulated data.** Metrics for niche identification (NID) and gene program recovery (GPR) across different model configurations and hyperparameters, based on n = 8 training runs per configuration, with varying random seeds. The top row in each panel shows the correspondence between predicted and ground truth niches; the bottom row contains F1 scores between enriched and artificially incremented programs and member genes (i.e. upregulated in the simulation) in ground truth niches. Boxplot elements are defined as: center line, median; box limits, upper and lower quartiles; whiskers, 1.5x interquartile range. The red dotted line marks the baseline F1 score between the artificially incremented programs and member genes and randomly selected candidate programs and genes (Methods; only programs and genes that could have been selected by NicheCompass as prior or *de novo* programs/genes, respectively, qualified for selection; to avoid score differences due to the number of programs and genes, the number of random programs and genes was matched for each run with the number of programs and member genes identified by NicheCompass and the average across all runs was reported). Ablations were performed on **a**, the weighting of the reconstruction losses indicating that both edge and gene expression reconstruction are essential components, **b**, the weighting of the regularization losses, indicating that regularization improves NID and is important to retrieve correct *de novo* genes, **c**, the encoder architecture, indicating that NicheCompass Light performs better on NID while NicheCompass has superior GPR on prior programs, **d**, the neighborhood size indicating better NID and retrieval of *de novo* genes for lower k and better GPR for higher k. GP: gene program.

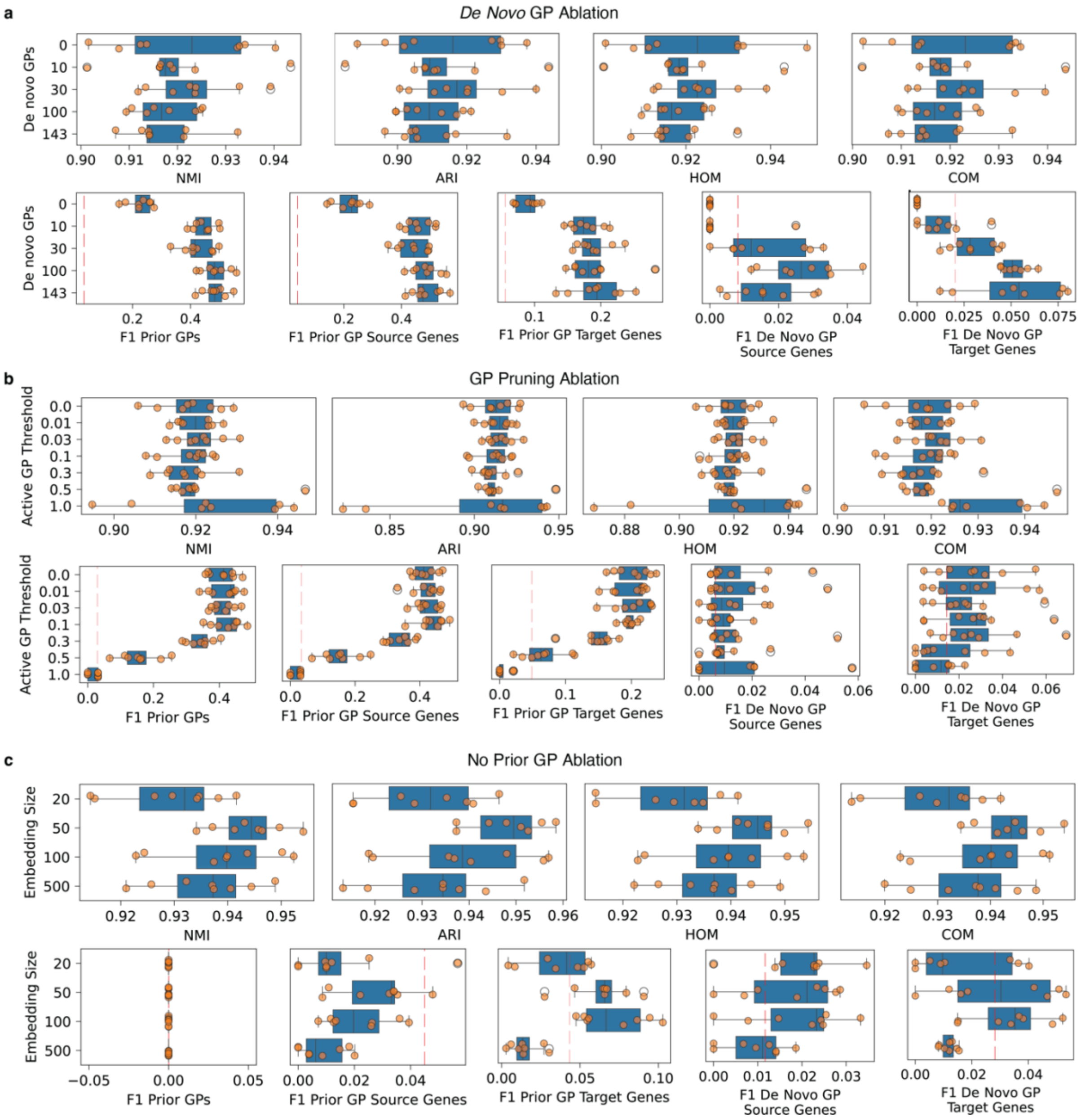

Supplementary Fig. 11 | Extended ablation study on simulated data. Metrics for niche identification (NID) and gene program recovery (GPR) across different model configurations and hyperparameters, based on n = 8 training runs per configuration, with varying random seeds. The top row in each panel shows the correspondence between predicted and ground truth niches; the bottom row contains F1 scores between enriched and artificially incremented programs and member genes (i.e. upregulated in the simulation) in ground truth niches. Boxplot elements are defined as: center line, median; box limits, upper and lower quartiles; whiskers, 1.5x interquartile range. The red dotted line marks the baseline F1 score between the artificially incremented programs and member genes and randomly selected candidate programs and genes (Methods; only programs and genes that could have been selected by NicheCompass as prior or *de novo* programs/genes, respectively, qualified for selection; to avoid score differences due to the number of programs and genes, the number of random programs and genes was matched for each run with the number of programs and member genes identified by NicheCompass and the average across all runs was reported). Ablations were performed on **a**, the number of *de novo* programs, indicating that the removal of *de novo* programs significantly deteriorates GPR performance, **b**, Program pruning via the active program threshold (Methods), indicating slightly improved NID and GPR under program pruning despite a reduced embedding size. **c**, a design without prior programs and different embedding sizes, indicating good NID performance at the cost of program gene recovery compared to a design with prior programs. GP: gene program.

a

GP Pruning & Regularization Program Effects

Active GP Thresh	Prior GP Reg	De novo GP Reg	Total GPs	Active GPs (Warm-up)	Active GPs (Final)
0.01	0.0	3.0	440	434	322
0.01	0.0	30.0	440	319	302
0.01	0.0	300.0	440	301	299
0.01	3.0	3.0	440	416	303
0.01	30.0	30.0	440	303	276
0.01	300.0	300.0	440	292	275
0.1	0.0	3.0	440	292	260
0.1	0.0	30.0	440	250	246
0.1	0.0	300.0	440	248	245
0.1	3.0	3.0	440	288	248
0.1	30.0	30.0	440	226	208
0.1	300.0	300.0	440	227	225

b

GP Pruning & Regularization Source Gene Effects

Active GP Thresh	Prior GP Reg	De novo GP Reg	Total Sources	Active Sources (Warm-up)	Active Sources (Final)
0.01	0.0	3.0	119547	106762	18107
0.01	0.0	30.0	119547	17076	4509
0.01	0.0	300.0	119547	3039	749
0.01	3.0	3.0	119547	105950	18322
0.01	30.0	30.0	119547	20304	4092
0.01	300.0	300.0	119547	3507	336
0.1	0.0	3.0	119547	35828	13332
0.1	0.0	30.0	119547	5522	3710
0.1	0.0	300.0	119547	1622	509
0.1	3.0	3.0	119547	42053	13209
0.1	30.0	30.0	119547	6095	3467
0.1	300.0	300.0	119547	1510	159

c

GP Pruning & Regularization Target Gene Effects

Active GP Thresh	Prior GP Reg	De novo GP Reg	Total Targets	Active Targets (Warm-up)	Active Targets (Final)
0.01	0.0	3.0	119687	112072	42372
0.01	0.0	30.0	119687	41839	32251
0.01	0.0	300.0	119687	30444	28739
0.01	3.0	3.0	119687	111288	42568
0.01	30.0	30.0	119687	44485	32101
0.01	300.0	300.0	119687	30951	28698
0.1	0.0	3.0	119687	55549	37809
0.1	0.0	30.0	119687	32155	30646
0.1	0.0	300.0	119687	28864	28071
0.1	3.0	3.0	119687	60469	37534
0.1	30.0	30.0	119687	32181	28490
0.1	300.0	300.0	119687	28865	27797

Supplementary Fig. 12 | Effect of program pruning and regularization. Number of total and active programs (**a**), program source (**b**), and program target genes (**c**) for different program pruning and gene weight regularization hyperparameters. GP: gene program.

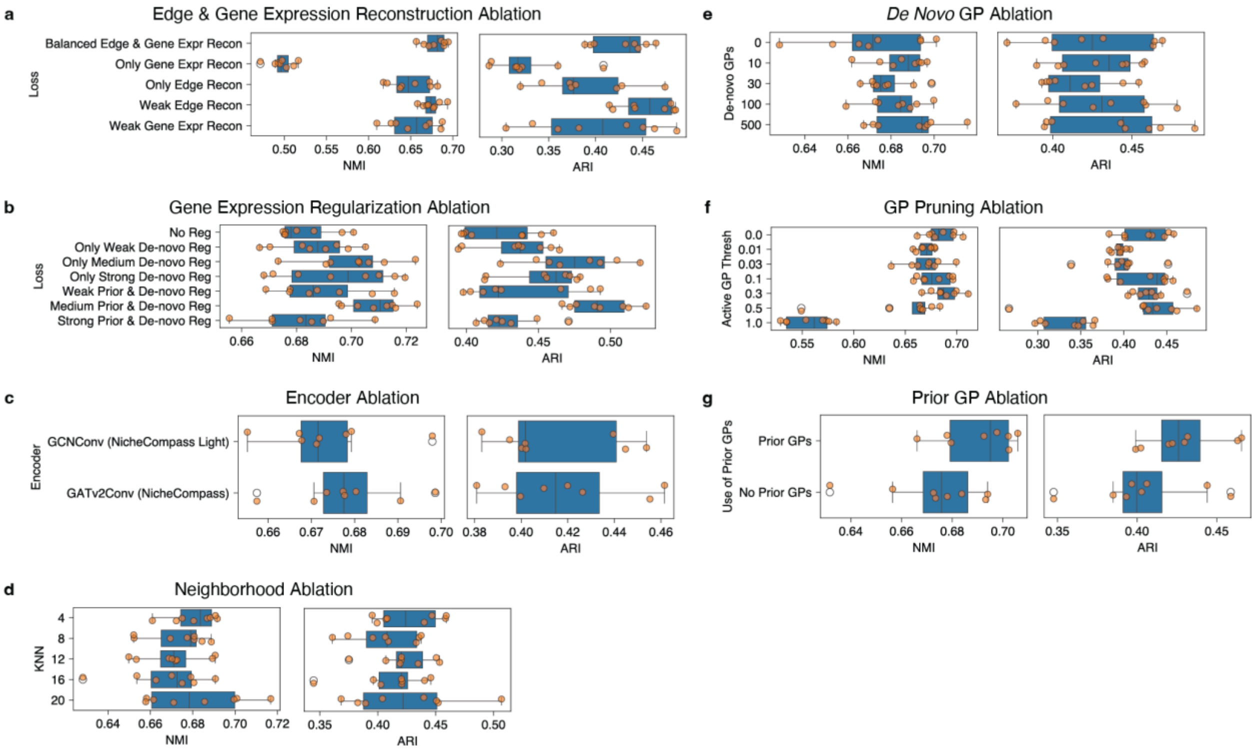

Supplementary Fig. 13 | Ablation study on real data. Niche identification (NID) metrics across different model configurations and hyperparameters, based on n = 8 training runs per configuration, with varying random seeds. Metrics measure the correspondence between predicted and ground truth niches (provided by the authors in the original study[11]). Boxplot elements are defined as: center line, median; box limits, upper and lower quartiles; whiskers, 1.5x interquartile range. Ablations were performed on **a**, the weighting of the reconstruction losses indicating that both edge and gene expression reconstruction are essential components, **b**, the weighting of the regularization losses, indicating that regularization improves NID. **c**, the encoder architecture, indicating that NicheCompass performs better on NID than NicheCompass Light, **d**, the neighborhood size indicating better NID for lower k. **e**, the number of *de novo* programs, indicating that inclusion of *de novo* programs slightly improves NID, **f**, Program pruning via the active program threshold (Methods), indicating that program pruning can slightly boost NID while reducing the model embedding size, and **g**, the use of prior programs, showing better NID when prior programs are used. GP: gene program.

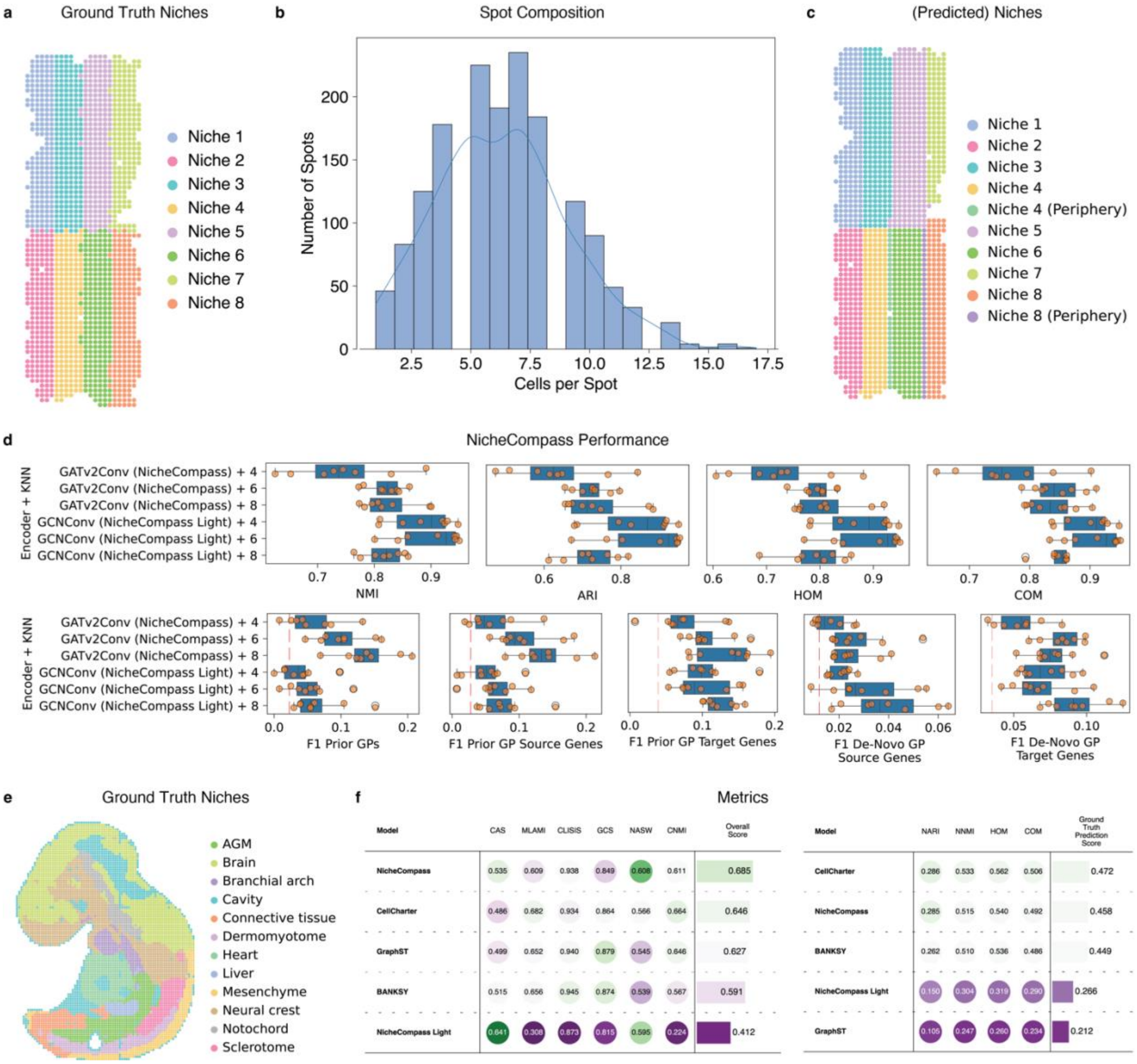

Supplementary Fig. 14 | **Ablation study and benchmarking on spot-level data. a**, Ground truth niches of single-cell resolution simulated data binned into spots with 55µm diameter. **b**, Distribution of the number of cells per bin/spot. **c**, Niches identified by NicheCompass, with misclassifications at the peripheries of Niches 4 and 8. **d**, Niche identification and gene program recovery metrics on binned simulation data for different encoder layer and k-nearest neighbors graph combinations across n = 8 training runs per combination, with varying random seeds. Boxplot elements are defined as: center line, median; box limits, upper and lower quartiles; whiskers, 1.5x interquartile range. **e**, Ground truth niches of the spot-level resolution Stereo-seq mouse embryo dataset[12]. **f**, Mean NicheCompass and ground truth prediction metrics across n = 8 training runs for each method, ranking NicheCompass first and second, respectively. STACI[13] encountered memory issues on our 40GB GPU, and DeepLinc[8] failed to converge; both were hence excluded. Overall and ground truth prediction scores were computed by aggregating the min-max-normalized individual metrics. GP: gene program.

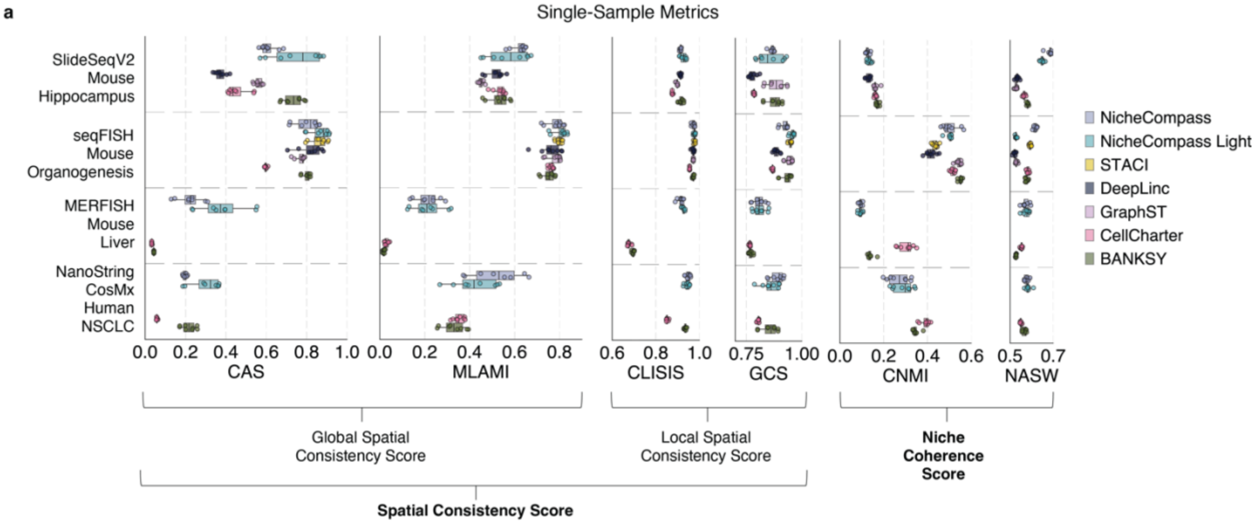

Supplementary Fig. 15 | **Single-sample benchmarking metrics. a**, Individual metrics from single-sample benchmarking across diverse datasets with n = 8 training runs per dataset and method, while varying sizes of the k-nearest neighbors graph (2 runs per k with k = 4, 8, 12, 16). Bars display the mean and error bars display the 95% confidence interval.

a Single-Sample Metric Averages: SlideSeqV2 Mouse Hippocampus

Model	SlideSeqV2 Mouse Hippocampus (100%) (0)						SlideSeqV2 Mouse Hippocampus (50%) (1)					
	CAS	MLAMI	CLISIS	GCS	NASW	CNMI	CAS	MLAMI	CLISIS	GCS	NASW	CNMI
BANKSY	0.732	0.532	0.916	0.875	0.581	0.171	0.805	0.506	0.921	0.889	0.606	0.217
NicheCompass	0.611	0.622	0.913	0.865	0.683	0.126	0.646	0.596	0.920	0.887	0.676	0.170
NicheCompass Light	0.752	0.572	0.926	0.857	0.648	0.133	0.777	0.461	0.920	0.812	0.661	0.182
GraphST	0.560	0.451	0.897	0.883	0.527	0.164	0.549	0.406	0.900	0.861	0.532	0.219
CellCharter	0.455	0.534	0.876	0.784	0.565	0.162	0.578	0.482	0.879	0.783	0.565	0.211
DeepLinc	0.374	0.516	0.912	0.778	0.533	0.130	0.455	0.523	0.914	0.796	0.538	0.180
STACI												

Model	SlideSeqV2 Mouse Hippocampus (25%) (2)						SlideSeqV2 Mouse Hippocampus (10%) (3)					
	CAS	MLAMI	CLISIS	GCS	NASW	CNMI	CAS	MLAMI	CLISIS	GCS	NASW	CNMI
BANKSY	0.869	0.545	0.928	0.908	0.604	0.235	0.914	0.642	0.942	0.922	0.579	0.275
NicheCompass	0.824	0.622	0.926	0.894	0.667	0.197	0.785	0.343	0.915	0.846	0.591	0.193
NicheCompass Light	0.837	0.485	0.921	0.820	0.666	0.209	0.856	0.486	0.925	0.848	0.636	0.233
GraphST	0.620	0.419	0.898	0.829	0.537	0.259	0.682	0.391	0.904	0.792	0.528	0.293
CellCharter	0.706	0.507	0.881	0.786	0.563	0.226	0.791	0.544	0.889	0.796	0.557	0.267
DeepLinc	0.568	0.556	0.916	0.808	0.533	0.194	0.672	0.626	0.922	0.818	0.521	0.208
STACI	0.892	0.659	0.947	0.925	0.565	0.165	0.924	0.786	0.963	0.952	0.557	0.126

Model	SlideSeqV2 Mouse Hippocampus (5%) (4)						SlideSeqV2 Mouse Hippocampus (1%) (5)					
	CAS	MLAMI	CLISIS	GCS	NASW	CNMI	CAS	MLAMI	CLISIS	GCS	NASW	CNMI
BANKSY	0.939	0.747	0.950	0.941	0.563	0.289	0.947	0.849	0.942	0.984	0.629	0.328
NicheCompass	0.809	0.359	0.906	0.851	0.641	0.185	0.894	0.565	0.911	0.913	0.753	0.299
NicheCompass Light	0.893	0.505	0.928	0.867	0.633	0.229	0.939	0.755	0.937	0.964	0.607	0.343
GraphST	0.763	0.377	0.895	0.779	0.522	0.342						
CellCharter	0.863	0.575	0.895	0.807	0.556	0.284	0.914	0.618	0.922	0.864	0.554	0.359
DeepLinc	0.786	0.722	0.930	0.834	0.518	0.205	0.864	0.741	0.900	0.862	0.514	0.217
STACI	0.915	0.872	0.958	0.962	0.551	0.126	0.838	0.825	0.912	0.972	0.629	0.223

Model	Aggregates					
	Overall Score (0)	Overall Score (1)	Overall Score (2)	Overall Score (3)	Overall Score (4)	Overall Score (5)
BANKSY	0.585	0.676	0.668	0.684	0.655	0.728
NicheCompass	0.583	0.637	0.684	0.398	0.380	0.574
NicheCompass Light	0.582	0.573	0.603	0.583	0.528	0.673
GraphST	0.371	0.389	0.387	0.349	0.320	
CellCharter	0.329	0.349	0.358	0.412	0.405	0.497
DeepLinc	0.210	0.288	0.293	0.326	0.342	0.273
STACI			0.561	0.560	0.516	0.474

Supplementary Fig. 16 | **Single-sample benchmarking on the SlideSeqV2 mouse hippocampus dataset. a**, Mean metrics across n = 8 training runs while varying sizes of the k-nearest neighbors graph (2 runs per k with k = 4, 8, 12, 16) for different subsample sizes of the SlideSeqV2 mouse hippocampus dataset[10]. Missing entries are due to memory overflow or failure of the model to converge.

a

Single-Sample Metric Averages: seqFISH Mouse Organogenesis

Model	seqFISH Mouse Organogenesis (100%) (0)						seqFISH Mouse Organogenesis (50%) (1)						seqFISH Mouse Organogenesis (25%) (2)						seqFISH Mouse Organogenesis (10%) (3)					
	CAS	MLAMI	CLISIS	GCS	NASW	CNMI	CAS	MLAMI	CLISIS	GCS	NASW	CNMI	CAS	MLAMI	CLISIS	GCS	NASW	CNMI	CAS	MLAMI	CLISIS	GCS	NASW	CNMI
NicheCompass	0.802	0.782	0.972	0.922	0.616	0.504	0.815	0.768	0.985	0.906	0.637	0.360	0.752	0.675	0.997	0.886	0.631	0.157	0.798	0.589	0.994	0.880	0.635	0.191
BANKSY	0.800	0.752	0.972	0.931	0.576	0.550	0.837	0.781	0.987	0.937	0.589	0.398	0.706	0.832	0.998	0.944	0.603	0.205	0.797	0.819	0.998	0.953	0.599	0.258
STACI	0.860	0.798	0.978	0.947	0.593	0.436	0.883	0.803	0.989	0.948	0.607	0.301	0.815	0.831	0.998	0.949	0.626	0.095	0.871	0.830	0.998	0.949	0.634	0.146
NicheCompass Light	0.867	0.806	0.977	0.956	0.528	0.501	0.892	0.799	0.989	0.957	0.540	0.377	0.821	0.837	0.998	0.957	0.552	0.168	0.849	0.810	0.997	0.957	0.576	0.218
GraphST	0.770	0.780	0.972	0.943	0.529	0.542	0.796	0.790	0.986	0.940	0.531	0.409	0.640	0.796	0.997	0.933	0.531	0.192	0.678	0.753	0.996	0.916	0.538	0.252
CellCharter	0.597	0.763	0.955	0.867	0.583	0.516	0.718	0.795	0.980	0.869	0.594	0.372	0.591	0.808	0.998	0.860	0.605	0.170	0.680	0.793	0.997	0.859	0.604	0.225
DeepLinc	0.819	0.758	0.971	0.885	0.520	0.416	0.765	0.761	0.983	0.875	0.526	0.323	0.627	0.774	0.997	0.874	0.534	0.137	0.654	0.736	0.994	0.865	0.534	0.159

Model	seqFISH Mouse Organogenesis (5%) (4)						seqFISH Mouse Organogenesis (1%) (5)						Aggregates					
	CAS	MLAMI	CLISIS	GCS	NASW	CNMI	CAS	MLAMI	CLISIS	GCS	NASW	CNMI	Overall Score (0)	Overall Score (1)	Overall Score (2)	Overall Score (3)	Overall Score (4)	Overall Score (5)
NicheCompass	0.751	0.553	0.991	0.892	0.646	0.211	0.833	0.370	0.983	0.877	0.739	0.266	0.718	0.631	0.616	0.519	0.550	0.576
BANKSY	0.788	0.884	0.995	0.960	0.582	0.300	0.819	0.914	0.995	0.977	0.618	0.325	0.682	0.682	0.774	0.750	0.722	0.736
STACI	0.854	0.787	0.994	0.948	0.654	0.129	0.828	0.757	0.991	0.932	0.712	0.271	0.669	0.611	0.679	0.678	0.641	0.687
NicheCompass Light	0.789	0.795	0.995	0.957	0.580	0.252	0.844	0.490	0.990	0.899	0.694	0.285	0.631	0.630	0.633	0.655	0.651	0.626
GraphST	0.583	0.624	0.992	0.887	0.533	0.293	0.740	0.286	0.992	0.848	0.515	0.294	0.593	0.574	0.517	0.499	0.435	0.370
CellCharter	0.626	0.705	0.994	0.846	0.588	0.248	0.798	0.453	0.992	0.868	0.544	0.308	0.425	0.473	0.585	0.565	0.479	0.479
DeepLinc	0.571	0.665	0.989	0.857	0.528	0.172	0.687	0.551	0.990	0.843	0.513	0.221	0.337	0.296	0.358	0.267	0.254	0.293

Supplementary Fig. 17 | Single-sample benchmarking on the seqFISH mouse organogenesis dataset. a, Mean metrics across n = 8 training runs while varying sizes of the k-nearest neighbors graph (2 runs per k with k = 4, 8, 12, 16) for different subsample sizes of the seqFISH mouse organogenesis dataset[1].

a

Single-Sample Metric Averages: MERFISH Mouse Liver

Model	MERFISH Mouse Liver (100%) (0)						MERFISH Mouse Liver (50%) (1)						MERFISH Mouse Liver (25%) (2)						MERFISH Mouse Liver (10%) (3)					
	CAS	MLAMI	CLISIS	GCS	NASW	CNMI	CAS	MLAMI	CLISIS	GCS	NASW	CNMI	CAS	MLAMI	CLISIS	GCS	NASW	CNMI	CAS	MLAMI	CLISIS	GCS	NASW	CNMI
NicheCompass Light	0.381	0.215	0.926	0.810	0.575	0.093	0.445	0.171	0.926	0.802	0.596	0.090	0.525	0.164	0.925	0.800	0.604	0.094	0.641	0.162	0.925	0.798	0.613	0.099
NicheCompass	0.223	0.214	0.913	0.807	0.572	0.094	0.199	0.151	0.892	0.789	0.601	0.092	0.253	0.164	0.885	0.786	0.608	0.093	0.367	0.185	0.881	0.792	0.618	0.097
CellCharter	0.033	0.030	0.677	0.765	0.553	0.303	0.047	0.032	0.684	0.764	0.553	0.293	0.066	0.042	0.705	0.766	0.548	0.307	0.107	0.068	0.725	0.769	0.557	0.282
BANKSY	0.043	0.018	0.696	0.770	0.528	0.139	0.062	0.024	0.704	0.771	0.532	0.139	0.090	0.033	0.717	0.775	0.533	0.157	0.148	0.058	0.734	0.782	0.530	0.156
DeepLinc																								
GraphST																			0.260	0.060	0.775	0.842	0.510	0.280
STACI																								

Model	MERFISH Mouse Liver (5%) (4)						MERFISH Mouse Liver (1%) (5)						Aggregates					
	CAS	MLAMI	CLISIS	GCS	NASW	CNMI	CAS	MLAMI	CLISIS	GCS	NASW	CNMI	Overall Score (0)	Overall Score (1)	Overall Score (2)	Overall Score (3)	Overall Score (4)	Overall Score (5)
NicheCompass Light	0.746	0.184	0.927	0.794	0.624	0.093	0.853	0.184	0.923	0.777	0.698	0.075	0.545	0.552	0.593	0.557	0.563	0.493
NicheCompass	0.447	0.213	0.876	0.792	0.631	0.093	0.557	0.250	0.841	0.809	0.644	0.089	0.488	0.468	0.503	0.498	0.508	0.385
CellCharter	0.152	0.089	0.715	0.772	0.558	0.320	0.344	0.143	0.732	0.780	0.531	0.316	0.324	0.316	0.301	0.322	0.357	0.286
BANKSY	0.217	0.076	0.744	0.789	0.532	0.137	0.483	0.187	0.788	0.827	0.515	0.137	0.098	0.116	0.131	0.171	0.177	0.225
DeepLinc																		
GraphST	0.356	0.079	0.778	0.848	0.510	0.270	0.556	0.131	0.783	0.851	0.515	0.291				0.350	0.353	0.377
STACI	0.734	0.173	0.914	0.882	0.528	0.100	0.863	0.315	0.931	0.885	0.562	0.092					0.455	0.497

Supplementary Fig. 18 | Single-sample benchmarking on the MERFISH mouse liver dataset. a, Mean metrics across n = 8 training runs while varying sizes of the k-nearest neighbors graph (2 runs per k with k = 4, 8, 12, 16) for different subsample sizes of the MERFISH mouse liver dataset. Missing entries are due to memory overflow or failure of the model to converge.

a

Single-Sample Metric Averages: nanoString CosMx Human NSCLC

Model	nanoString CosMx Human NSCLC (100%) (0)						nanoString CosMx Human NSCLC (50%) (1)						nanoString CosMx Human NSCLC (25%) (2)						nanoString CosMx Human NSCLC (10%) (3)					
	CAS	MLAMI	CLISIS	GCS	NASW	CNMI	CAS	MLAMI	CLISIS	GCS	NASW	CNMI	CAS	MLAMI	CLISIS	GCS	NASW	CNMI	CAS	MLAMI	CLISIS	GCS	NASW	CNMI
**NicheCompass Light**	0.302	0.424	0.943	0.859	0.583	0.289	0.454	0.429	0.929	0.843	0.591	0.287	0.609	0.399	0.921	0.811	0.609	0.252	0.701	0.407	0.921	0.840	0.631	0.243
**NicheCompass**	0.197	0.519	0.948	0.888	0.576	0.267	0.339	0.560	0.939	0.885	0.599	0.236	0.580	0.588	0.936	0.886	0.620	0.214	0.763	0.603	0.929	0.886	0.602	0.237
**BANKSY**	0.216	0.329	0.935	0.854	0.567	0.346	0.359	0.382	0.919	0.866	0.567	0.325	0.559	0.409	0.914	0.882	0.559	0.277	0.746	0.544	0.922	0.915	0.546	0.275
**CellCharter**	0.059	0.352	0.852	0.803	0.550	0.390	0.085	0.374	0.780	0.802	0.548	0.390	0.135	0.337	0.737	0.794	0.561	0.367	0.286	0.400	0.808	0.799	0.560	0.348
**DeepLinc**							0.111	0.297	0.925	0.804	0.506	0.207	0.183	0.317	0.918	0.816	0.514	0.187	0.288	0.370	0.921	0.816	0.519	0.204
**GraphST**							0.156	0.351	0.891	0.914	0.530	0.379	0.257	0.345	0.879	0.908	0.542	0.331	0.407	0.369	0.896	0.900	0.549	0.309
**STACI**																			0.763	0.587	0.934	0.916	0.552	0.215

Model	nanoString CosMx Human NSCLC (5%) (4)						nanoString CosMx Human NSCLC (1%) (5)						Aggregates					
	CAS	MLAMI	CLISIS	GCS	NASW	CNMI	CAS	MLAMI	CLISIS	GCS	NASW	CNMI	Overall Score (0)	Overall Score (1)	Overall Score (2)	Overall Score (3)	Overall Score (4)	Overall Score (5)
**NicheCompass Light**	0.769	0.535	0.927	0.898	0.590	0.210	0.762	0.240	0.937	0.805	0.714	0.258	0.580	0.636	0.591	0.564	0.562	0.552
**NicheCompass**	0.812	0.581	0.926	0.885	0.606	0.236	0.771	0.428	0.941	0.874	0.649	0.237	0.548	0.651	0.689	0.616	0.623	0.553
**BANKSY**	0.845	0.633	0.929	0.940	0.547	0.283	0.813	0.779	0.953	0.968	0.585	0.289	0.503	0.598	0.559	0.574	0.637	0.673
**CellCharter**	0.432	0.362	0.818	0.798	0.564	0.365	0.619	0.309	0.905	0.813	0.552	0.355	0.290	0.405	0.373	0.370	0.383	0.366
**DeepLinc**	0.405	0.386	0.919	0.825	0.519	0.219	0.461	0.293	0.913	0.809	0.499	0.184		0.235	0.251	0.239	0.277	0.153
**GraphST**	0.517	0.335	0.892	0.875	0.551	0.331	0.558	0.193	0.909	0.820	0.518	0.324		0.533	0.499	0.474	0.463	0.285
**STACI**	0.871	0.684	0.945	0.935	0.568	0.197	0.759	0.806	0.954	0.948	0.566	0.098				0.537	0.615	0.497

Supplementary Fig. 19 | **Single-sample benchmarking on the NanoString CosMx human NSCLC dataset. a**, Mean metrics across n = 8 training runs while varying sizes of the k-nearest neighbors graph (2 runs per k with k = 4, 8, 12, 16) for different subsample sizes of the NanoString CosMx human NSCLC dataset[14]. Missing entries are due to memory overflow.

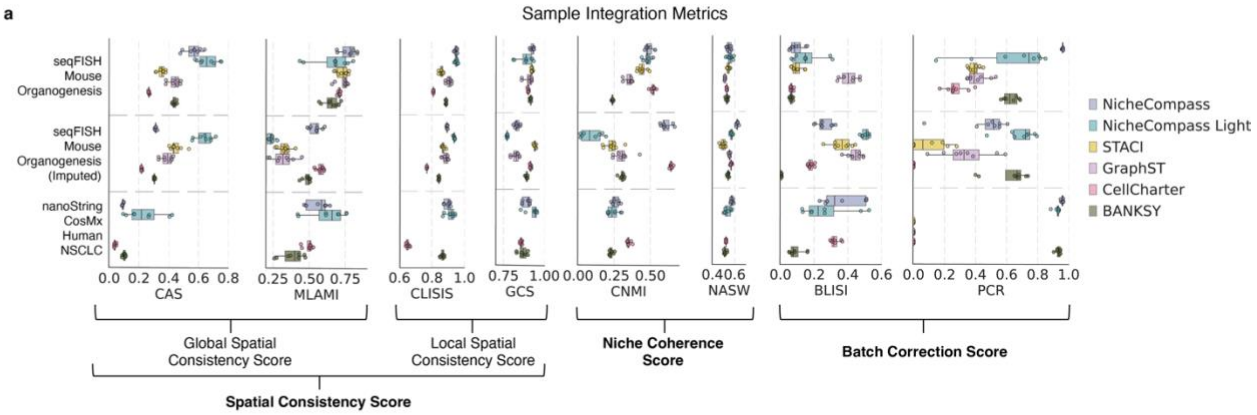

Supplementary Fig. 20 | **Sample integration benchmarking metrics. a**, Individual metrics from sample integration benchmarking across diverse datasets with n = 8 training runs per dataset and method, while varying sizes of the k-nearest neighbors graph (2 runs per k with k = 4, 8, 12, 16). Bars display the mean and error bars display the 95% confidence interval.

a

Sample Integration Metric Averages: seqFISH Mouse Organogenesis

	seqFISH Mouse Organogenesis (100%) (0)								seqFISH Mouse Organogenesis (50%) (1)								seqFISH Mouse Organogenesis (25%) (2)								seqFISH Mouse Organogenesis (10%) (3)							
**Model**	CAS	MLAMI	CLISIS	GCS	NASW	CNMI	BLISI	PCR	CAS	MLAMI	CLISIS	GCS	NASW	CNMI	BLISI	PCR	CAS	MLAMI	CLISIS	GCS	NASW	CNMI	BLISI	PCR	CAS	MLAMI	CLISIS	GCS	NASW	CNMI	BLISI	PCR
**NicheCompass**	0.564	0.772	0.947	0.926	0.548	0.491	0.095	0.961	0.687	0.784	0.957	0.926	0.557	0.434	0.089	0.000	0.753	0.749	0.968	0.912	0.563	0.376	0.085	0.000	0.846	0.621	0.960	0.892	0.588	0.408	0.098	0.000
**NicheCompass Light**	0.662	0.657	0.950	0.883	0.558	0.481	0.160	0.634	0.792	0.653	0.960	0.884	0.540	0.401	0.141	0.000	0.840	0.592	0.971	0.880	0.523	0.333	0.120	0.000	0.911	0.619	0.969	0.910	0.509	0.387	0.079	0.000
**GraphST**	0.438	0.734	0.905	0.903	0.511	0.351	0.405	0.413	0.501	0.761	0.907	0.903	0.511	0.323	0.382	0.000	0.598	0.764	0.923	0.904	0.513	0.317	0.330	0.000	0.744	0.670	0.919	0.900	0.517	0.381	0.307	0.000
**STACI**	0.351	0.729	0.858	0.924	0.543	0.444	0.097	0.391	0.614	0.767	0.922	0.932	0.547	0.466	0.098	0.557	0.751	0.697	0.950	0.932	0.528	0.349	0.092	0.671	0.853	0.581	0.938	0.926	0.548	0.361	0.105	0.650
**GraphST (No Prior Alignment)**	0.581	0.767	0.948	0.946	0.507	0.500	0.074	0.000	0.692	0.801	0.958	0.946	0.514	0.506	0.066	0.000	0.739	0.788	0.970	0.943	0.521	0.475	0.058	0.000	0.836	0.720	0.961	0.938	0.536	0.525	0.059	0.000
**CellCharter**	0.267	0.709	0.809	0.901	0.529	0.519	0.066	0.273	0.446	0.755	0.877	0.908	0.549	0.489	0.090	0.450	0.544	0.724	0.926	0.911	0.563	0.435	0.099	0.752	0.731	0.713	0.916	0.912	0.562	0.495	0.096	0.858
**BANKSY**	0.438	0.652	0.887	0.915	0.530	0.238	0.064	0.626	0.715	0.674	0.917	0.926	0.550	0.210	0.106	0.916	0.673	0.667	0.913	0.935	0.572	0.120	0.163	0.878	0.747	0.660	0.889	0.944	0.579	0.184	0.233	0.930

	seqFISH Mouse Organogenesis (5%) (4)								seqFISH Mouse Organogenesis (1%) (5)								Aggregates					
**Model**	CAS	MLAMI	CLISIS	GCS	NASW	CNMI	BLISI	PCR	CAS	MLAMI	CLISIS	GCS	NASW	CNMI	BLISI	PCR	Overall Score (0)	Overall Score (1)	Overall Score (2)	Overall Score (3)	Overall Score (4)	Overall Score (5)
**NicheCompass**	0.863	0.627	0.945	0.884	0.615	0.470	0.117	0.000	0.841	0.406	0.931	0.892	0.679	0.521	0.111	0.000	0.673	0.521	0.497	0.454	0.472	0.468
**NicheCompass Light**	0.919	0.601	0.959	0.890	0.541	0.417	0.098	0.000	0.898	0.634	0.956	0.921	0.621	0.533	0.098	0.000	0.624	0.470	0.405	0.365	0.356	0.480
**GraphST**	0.801	0.593	0.912	0.905	0.528	0.443	0.264	0.000	0.773	0.310	0.932	0.880	0.564	0.604	0.224	0.000	0.497	0.418	0.433	0.380	0.365	0.387
**STACI**	0.879	0.646	0.935	0.926	0.573	0.386	0.130	0.606	0.827	0.575	0.915	0.928	0.663	0.405	0.214	0.610	0.448	0.526	0.472	0.413	0.459	0.568
**GraphST (No Prior Alignment)**	0.860	0.762	0.941	0.932	0.548	0.601	0.067	0.000	0.813	0.337	0.936	0.905	0.579	0.651	0.104	0.000	0.445	0.503	0.504	0.465	0.445	0.412
**CellCharter**	0.798	0.756	0.910	0.915	0.562	0.555	0.137	0.880	0.748	0.432	0.915	0.934	0.537	0.516	0.312	0.871	0.373	0.409	0.515	0.533	0.574	0.545
**BANKSY**	0.731	0.718	0.862	0.957	0.589	0.177	0.301	0.918	0.620	0.679	0.732	0.966	0.599	0.191	0.582	0.965	0.351	0.510	0.505	0.516	0.550	0.563

Supplementary Fig. 21 | **Sample integration benchmarking on the seqFISH mouse organogenesis dataset. a**, Mean metrics across n = 8 training runs while varying sizes of the k-nearest neighbors graph (2 runs per k with k = 4, 8, 12, 16) for different subsample sizes of the seqFISH mouse organogenesis dataset[1].

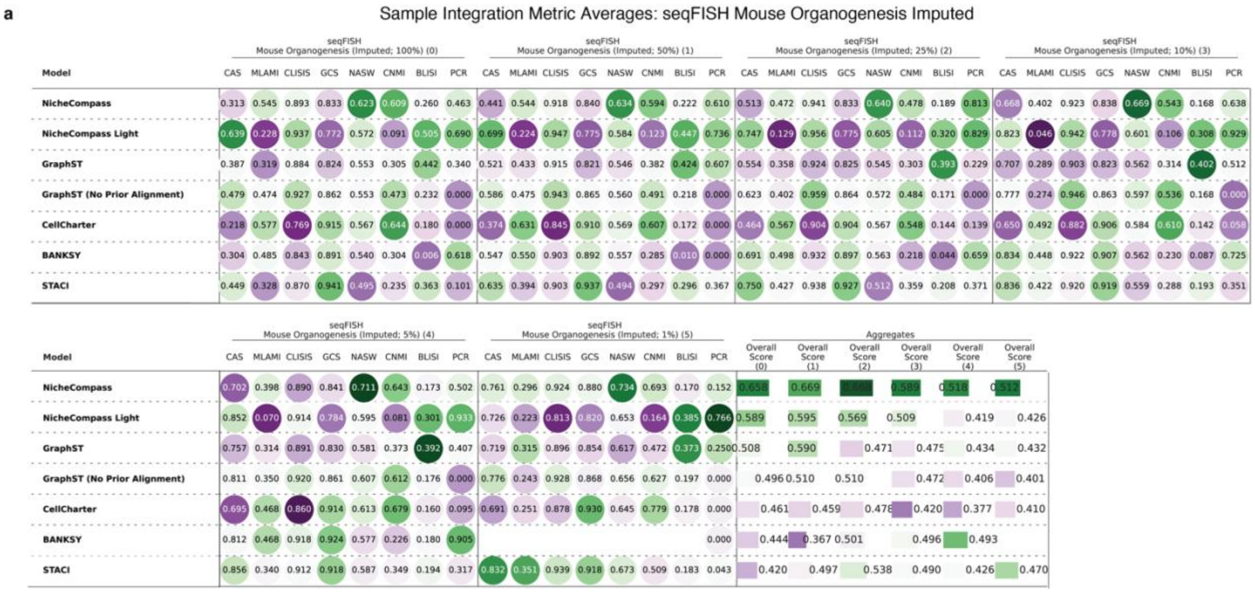

a

Sample Integration Metric Averages: seqFISH Mouse Organogenesis Imputed

Model	seqFISH Mouse Organogenesis (Imputed; 100%) (0)								seqFISH Mouse Organogenesis (Imputed; 50%) (1)							
	CAS	MLAMI	CLISIS	GCS	NASW	CNMI	BLISI	PCR	CAS	MLAMI	CLISIS	GCS	NASW	CNMI	BLISI	PCR
**NicheCompass**	0.313	0.545	0.893	0.833	0.623	0.609	0.260	0.463	0.441	0.544	0.918	0.840	0.634	0.594	0.222	0.610
**NicheCompass Light**	0.639	0.228	0.937	0.772	0.572	0.091	0.505	0.690	0.699	0.224	0.947	0.775	0.584	0.123	0.447	0.736
**GraphST**	0.387	0.319	0.884	0.824	0.553	0.305	0.442	0.340	0.521	0.433	0.915	0.821	0.546	0.382	0.424	0.607
**GraphST (No Prior Alignment)**	0.479	0.474	0.927	0.862	0.553	0.473	0.232	0.000	0.586	0.475	0.943	0.865	0.560	0.491	0.218	0.000
**CellCharter**	0.218	0.577	0.769	0.915	0.567	0.644	0.180	0.000	0.374	0.631	0.845	0.910	0.569	0.607	0.172	0.000
**BANKSY**	0.304	0.485	0.843	0.891	0.540	0.304	0.006	0.618	0.547	0.550	0.903	0.892	0.557	0.285	0.010	0.000
**STACI**	0.449	0.328	0.870	0.941	0.495	0.235	0.363	0.101	0.635	0.394	0.903	0.937	0.494	0.297	0.296	0.367

Model	seqFISH Mouse Organogenesis (Imputed; 25%) (2)								seqFISH Mouse Organogenesis (Imputed; 10%) (3)							
	CAS	MLAMI	CLISIS	GCS	NASW	CNMI	BLISI	PCR	CAS	MLAMI	CLISIS	GCS	NASW	CNMI	BLISI	PCR
**NicheCompass**	0.513	0.472	0.941	0.833	0.640	0.478	0.189	0.813	0.668	0.402	0.923	0.838	0.669	0.543	0.168	0.638
**NicheCompass Light**	0.747	0.129	0.956	0.775	0.605	0.112	0.320	0.829	0.823	0.046	0.942	0.778	0.601	0.106	0.308	0.929
**GraphST**	0.554	0.358	0.924	0.825	0.545	0.303	0.393	0.229	0.707	0.289	0.903	0.823	0.562	0.314	0.402	0.512
**GraphST (No Prior Alignment)**	0.623	0.402	0.959	0.864	0.572	0.484	0.171	0.000	0.777	0.274	0.946	0.863	0.597	0.536	0.168	0.000
**CellCharter**	0.464	0.567	0.904	0.904	0.567	0.548	0.144	0.139	0.650	0.492	0.882	0.906	0.584	0.610	0.142	0.058
**BANKSY**	0.691	0.498	0.932	0.897	0.563	0.218	0.044	0.659	0.834	0.448	0.922	0.907	0.562	0.230	0.087	0.725
**STACI**	0.750	0.427	0.938	0.927	0.512	0.359	0.208	0.371	0.836	0.422	0.920	0.919	0.559	0.288	0.193	0.351

Model	seqFISH Mouse Organogenesis (Imputed; 5%) (4)								seqFISH Mouse Organogenesis (Imputed; 1%) (5)							
	CAS	MLAMI	CLISIS	GCS	NASW	CNMI	BLISI	PCR	CAS	MLAMI	CLISIS	GCS	NASW	CNMI	BLISI	PCR
**NicheCompass**	0.702	0.398	0.890	0.841	0.711	0.643	0.173	0.502	0.761	0.296	0.924	0.880	0.734	0.693	0.170	0.152
**NicheCompass Light**	0.852	0.070	0.914	0.784	0.595	0.081	0.301	0.933	0.726	0.223	0.813	0.820	0.653	0.164	0.385	0.766
**GraphST**	0.757	0.314	0.891	0.830	0.581	0.373	0.392	0.407	0.719	0.315	0.896	0.854	0.617	0.472	0.373	0.250
**GraphST (No Prior Alignment)**	0.811	0.350	0.920	0.861	0.607	0.612	0.176	0.000	0.776	0.243	0.928	0.868	0.656	0.627	0.197	0.000
**CellCharter**	0.695	0.468	0.860	0.914	0.613	0.679	0.160	0.095	0.691	0.251	0.878	0.930	0.645	0.779	0.178	0.000
**BANKSY**	0.812	0.468	0.918	0.924	0.577	0.226	0.180	0.905								0.000
**STACI**	0.856	0.340	0.912	0.918	0.587	0.349	0.194	0.317	0.832	0.351	0.939	0.918	0.673	0.509	0.183	0.043

Model	Aggregates					
	Overall Score (0)	Overall Score (1)	Overall Score (2)	Overall Score (3)	Overall Score (4)	Overall Score (5)
**NicheCompass**	0.658	0.669	[illegible]	0.589	0.518	0.512
**NicheCompass Light**	0.589	0.595	0.569	0.509	0.419	0.426
**GraphST**	0.508	0.590	0.471	0.475	0.434	0.432
**GraphST (No Prior Alignment)**	0.496	0.510	0.510	0.472	0.406	0.401
**CellCharter**	0.461	0.459	0.478	0.420	0.377	0.410
**BANKSY**	0.444	0.367	0.501	0.496	0.493	
**STACI**	0.420	0.497	0.538	0.490	0.426	0.470

Supplementary Fig. 22 | **Sample integration benchmarking on the seqFISH mouse organogenesis imputed dataset. a**, Mean metrics across n = 8 training runs while varying sizes of the k-nearest neighbors graph (2 runs per k with k = 4, 8, 12, 16) for different subsample sizes of the seqFISH mouse organogenesis imputed dataset[1]. Missing entries are due to failure of the model to converge.

a

Sample Integration Metric Averages: nanoString Cosmx Human NSCLC

Model	nanoString CosMx Human NSCLC (100%) (0)							
	CAS	MLAMI	CLISIS	GCS	NASW	CNMI	BLISI	PCR
NicheCompass	0.092	0.558	0.895	0.891	0.575	0.258	0.368	0.959
NicheCompass Light	0.238	0.625	0.914	0.928	0.536	0.243	0.270	0.926
BANKSY	0.101	0.382	0.865	0.868	0.513	0.229	0.091	0.933
CellCharter	0.039	0.495	0.650	0.857	0.522	0.351	0.324	0.000
STACI								0.000
GraphST								0.000
GraphST (No Prior Alignment)								0.000

Model	nanoString CosMx Human NSCLC (50%) (1)							
	CAS	MLAMI	CLISIS	GCS	NASW	CNMI	BLISI	PCR
NicheCompass	0.115	0.536	0.901	0.892	0.555	0.288	0.347	0.970
NicheCompass Light	0.285	0.610	0.917	0.927	0.529	0.275	0.259	0.939
BANKSY	0.184	0.408	0.882	0.870	0.533	0.254	0.112	0.962
CellCharter	0.052	0.502	0.665	0.853	0.524	0.362	0.331	0.000
STACI								0.000
GraphST								0.000
GraphST (No Prior Alignment)								0.000

Model	nanoString CosMx Human NSCLC (25%) (2)							
	CAS	MLAMI	CLISIS	GCS	NASW	CNMI	BLISI	PCR
NicheCompass	0.172	0.555	0.913	0.894	0.561	0.283	0.256	0.974
NicheCompass Light	0.242	0.495	0.902	0.878	0.564	0.313	0.338	0.931
BANKSY	0.299	0.449	0.910	0.878	0.535	0.262	0.136	0.815
CellCharter	0.066	0.580	0.722	0.848	0.525	0.339	0.335	0.226
STACI								0.000
GraphST								0.000
GraphST (No Prior Alignment)								0.000

Model	nanoString CosMx Human NSCLC (10%) (3)							
	CAS	MLAMI	CLISIS	GCS	NASW	CNMI	BLISI	PCR
NicheCompass	0.272	0.561	0.897	0.886	0.596	0.292	0.410	0.957
NicheCompass Light	0.497	0.556	0.915	0.881	0.597	0.288	0.315	0.854
BANKSY	0.461	0.458	0.919	0.895	0.544	0.242	0.210	0.828
CellCharter	0.096	0.529	0.703	0.855	0.535	0.334	0.489	0.414
STACI	0.553	0.535	0.921	0.911	0.528	0.219	0.260	0.346
GraphST	0.175	0.434	0.839	0.917	0.518	0.349	0.299	0.000
GraphST (No Prior Alignment)	0.175	0.433	0.840	0.918	0.523	0.357	0.275	0.000

Model	nanoString CosMx Human NSCLC (5%) (4)							
	CAS	MLAMI	CLISIS	GCS	NASW	CNMI	BLISI	PCR
NicheCompass	0.459	0.691	0.916	0.902	0.583	0.232	0.255	0.968
NicheCompass Light	0.628	0.577	0.916	0.846	0.594	0.247	0.270	0.805
BANKSY	0.581	0.577	0.927	0.922	0.536	0.206	0.295	0.926
CellCharter	0.130	0.541	0.670	0.856	0.547	0.341	0.560	0.763
STACI	0.648	0.616	0.922	0.915	0.525	0.225	0.202	0.555
GraphST	0.242	0.462	0.840	0.896	0.537	0.297	0.435	0.040
GraphST (No Prior Alignment)	0.250	0.530	0.838	0.913	0.528	0.301	0.213	0.000

Model	nanoString CosMx Human NSCLC (1%) (5)							
	CAS	MLAMI	CLISIS	GCS	NASW	CNMI	BLISI	PCR
NicheCompass	0.703	0.491	0.904	0.877	0.587	0.187	0.184	0.859
NicheCompass Light	0.795	0.245	0.912	0.821	0.608	0.170	0.130	0.695
BANKSY								0.000
CellCharter	0.371	0.369	0.717	0.860	0.545	0.378	0.691	0.956
STACI	0.826	0.502	0.923	0.888	0.572	0.167	0.183	0.508
GraphST	0.466	0.226	0.809	0.831	0.537	0.284	0.486	0.243
GraphST (No Prior Alignment)	0.475	0.277	0.814	0.856	0.537	0.303	0.148	0.000

Model	Aggregates					
	Overall Score (0)	Overall Score (1)	Overall Score (2)	Overall Score (3)	Overall Score (4)	Overall Score (5)
NicheCompass	0.637	0.595	0.589	0.709	0.604	0.564
NicheCompass Light	0.583	0.579	0.642	0.680	0.575	0.487
BANKSY	0.345	0.418	0.429	0.488	0.514	
CellCharter	0.338	0.327	0.340	0.475	0.530	0.602
STACI				0.423	0.427	0.511
GraphST				0.379	0.379	0.384
GraphST (No Prior Alignment)				0.385	0.311	0.298

Supplementary Fig. 23 | Sample integration benchmarking on the NanoString CosMx human NSCLC dataset. a, Mean metrics across n = 8 training runs while varying sizes of the k-nearest neighbors graph (2 runs per k with k = 4, 8, 12, 16) for different subsample sizes of the nanoString CosMx human NSCLC dataset[14]. Missing entries are due to memory overflow or failure of the model to converge.

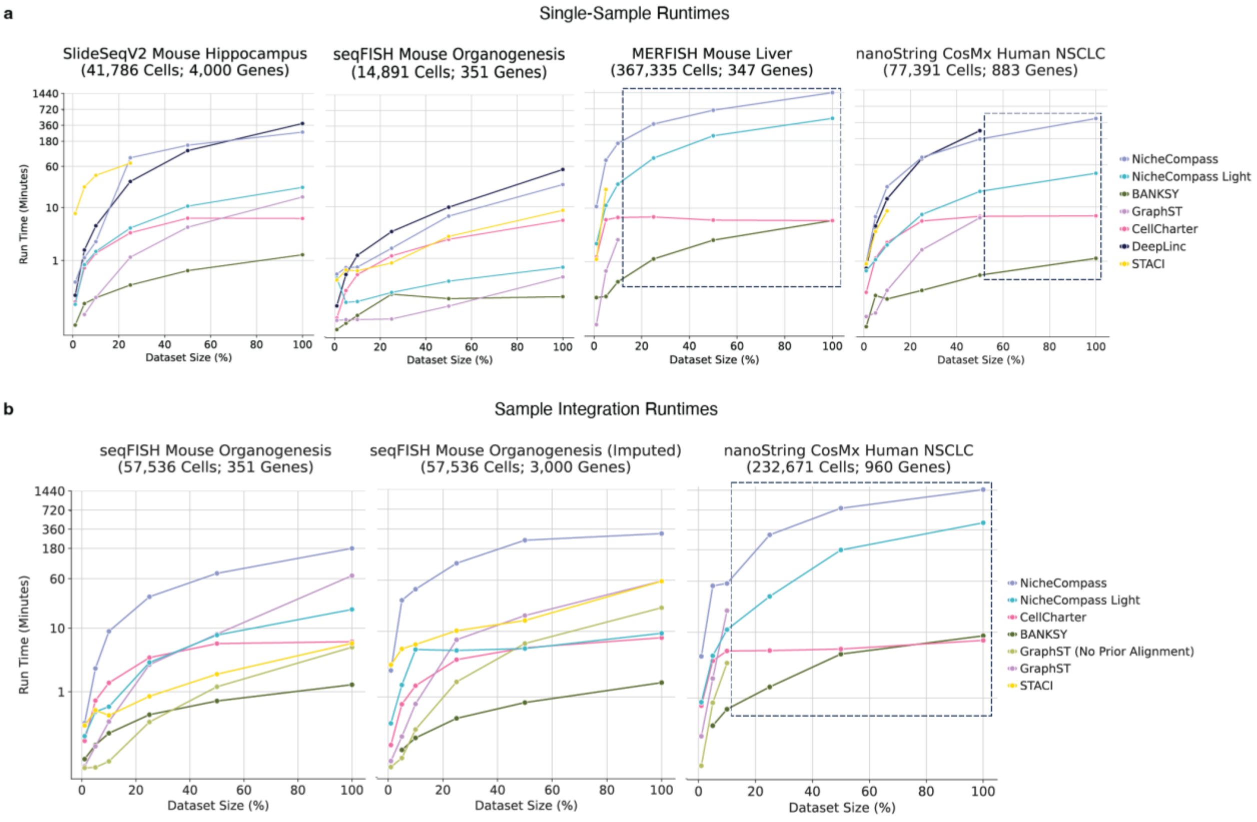

c NicheCompass: Single-Sample Model Sizes

Dataset	Number of Genes	Number of GPs	Number of Params
SlideSeqV2 Mouse Hippocampus	4000	1529	55802739
seqFISH Mouse Organogenesis	351	1368	8802846
MERFISH Mouse Liver	347	1388	8826986
Nanostring CosMx Human NSCLC	960	1325	23854930

d NicheCompass: Sample Integration Model Sizes

Dataset	Number of Genes	Number of GPs	Number of Params
seqFISH Mouse Organogenesis	351	1368	8804970
seqFISH Mouse Organogenesis (Imputed)	3000	1489	48939157
Nanostring CosMx Human NSCLC	960	1504	26958473

Supplementary Fig. 24 | **Benchmarking runtimes and model sizes. a**, Mean runtimes across n = 8 training runs per dataset and method trained during single-sample benchmarking, while varying sizes of the k-nearest neighbors graph (2 runs per k with k = 4, 8, 12, 16). Highlighted is the regime where only NicheCompass, CellCharter[7], and BANKSY[5] ran successfully. **b**, Same as **a** but for models trained during sample integration benchmarking. **c**, Overview of NicheCompass model sizes for datasets used for single-sample benchmarking, showing the impact of the number of genes on the number of programs and model parameters. **d**, Same as **c** but for datasets used for sample integration benchmarking. GP: gene program.

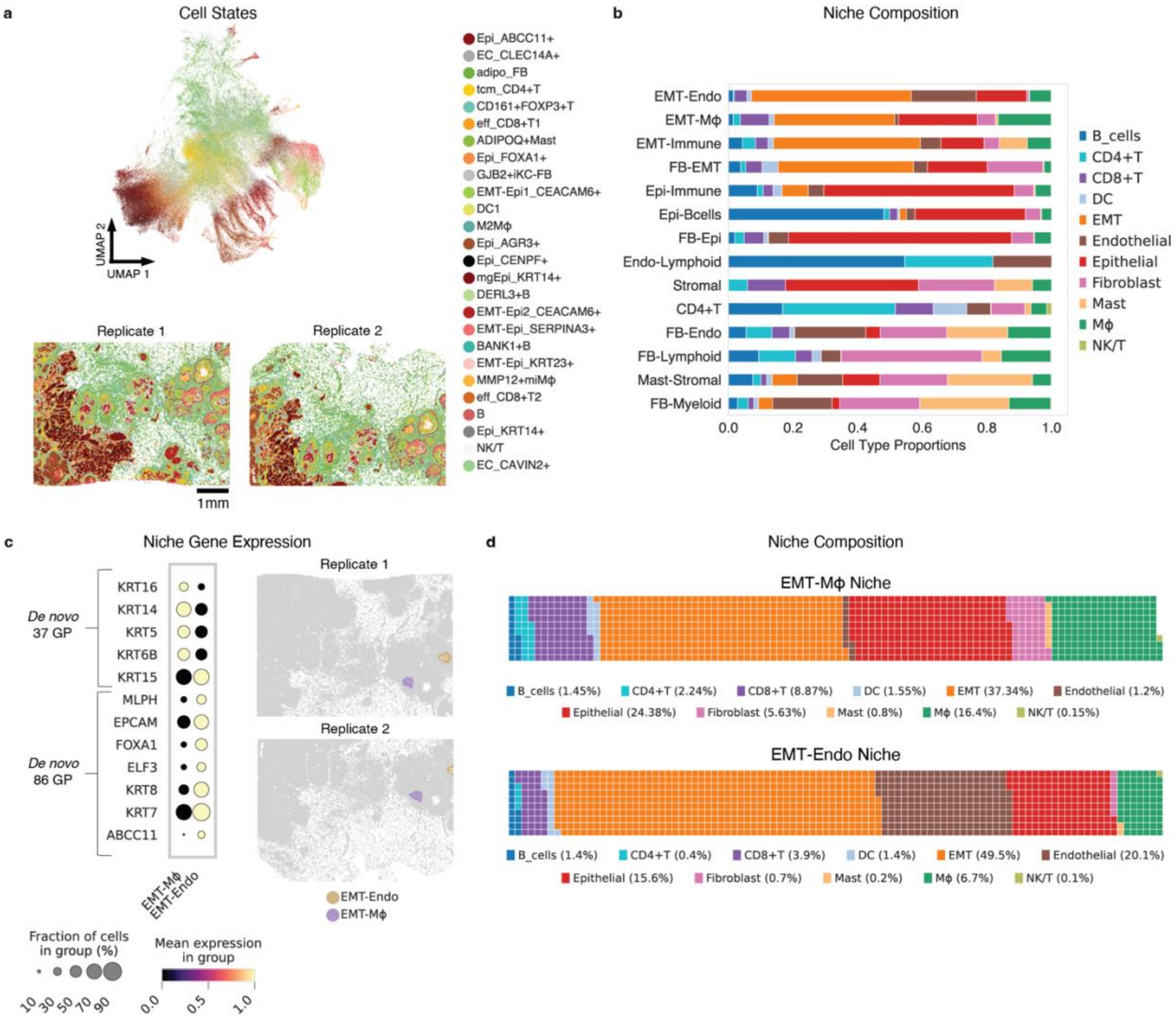

Supplementary Fig. 25 | **Extended Xenium human breast cancer analysis. a**, UMAP representation of the integrated NicheCompass embedding space and the two integrated tissue replicates[15], colored by annotated cell states. **b**, Cell type composition of all niches. **c**, Niche-specific normalized expression of important member genes of *de novo* 37 and *de novo* 86 programs, showing a clear separation between the two niches based on *KRT14* (basal breast tumor cells) and *KRT8* (luminal breast tumor cells) member gene transcriptional activities. Dot size indicates the fraction of cells in each niche with non-zero expression, and dot color represents the mean expression level among expressing cells, normalized across the two niches. **d**, Cellular composition of the EMT-Macrophage and EMT-Endo niches. GP: gene program.

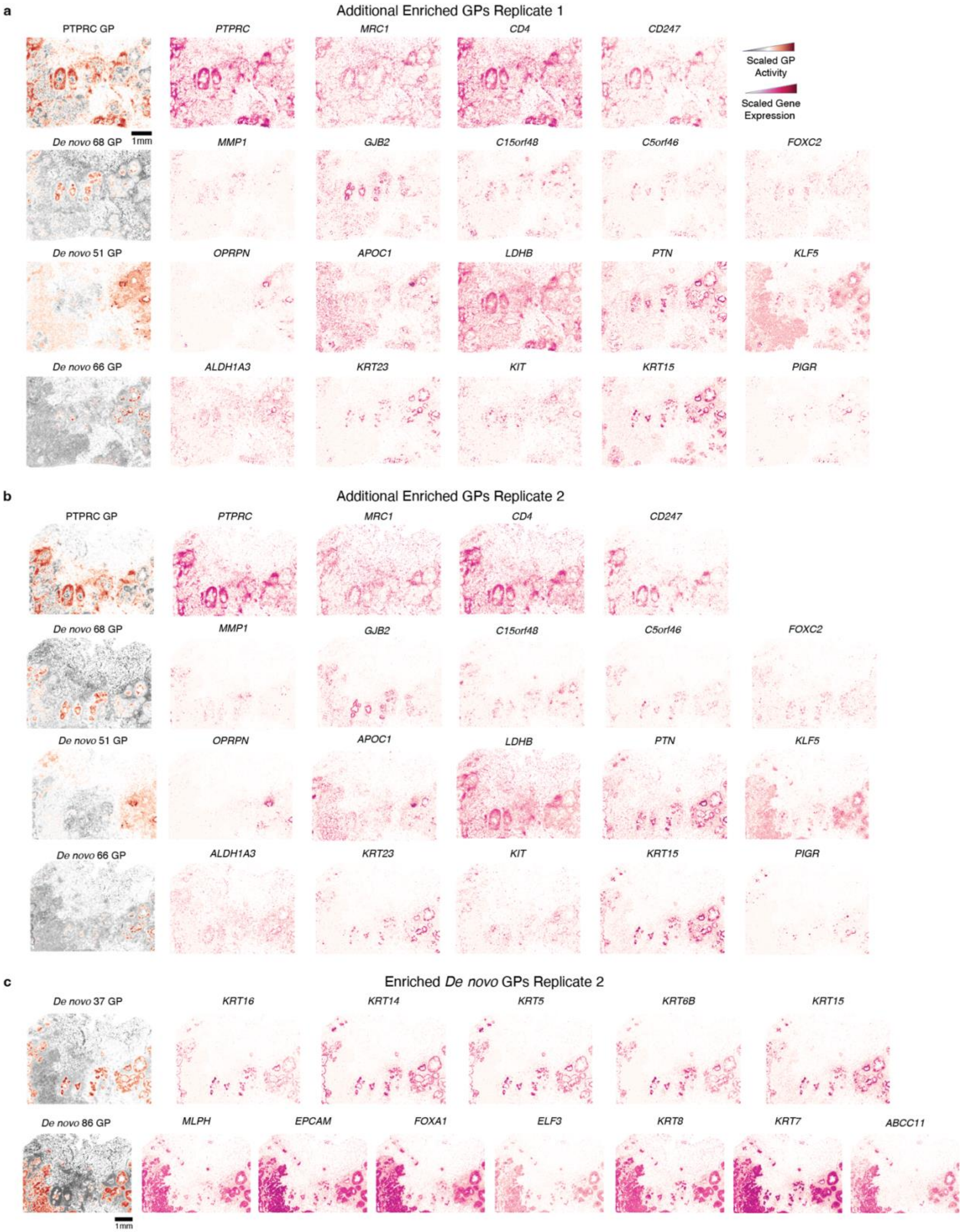

Supplementary Fig. 26 | **Additional enriched programs in the Xenium human breast cancer dataset.** **a**,**b**, Program activity and gene expression of the most important genes of additional niche-enriched programs in replicate 1 (**a**) and replicate 2 (**b**). **c**, Program activity and gene expression of the most important genes of enriched *de novo* 37 program and *de novo* 86 program in replicate 2. GP: gene program.

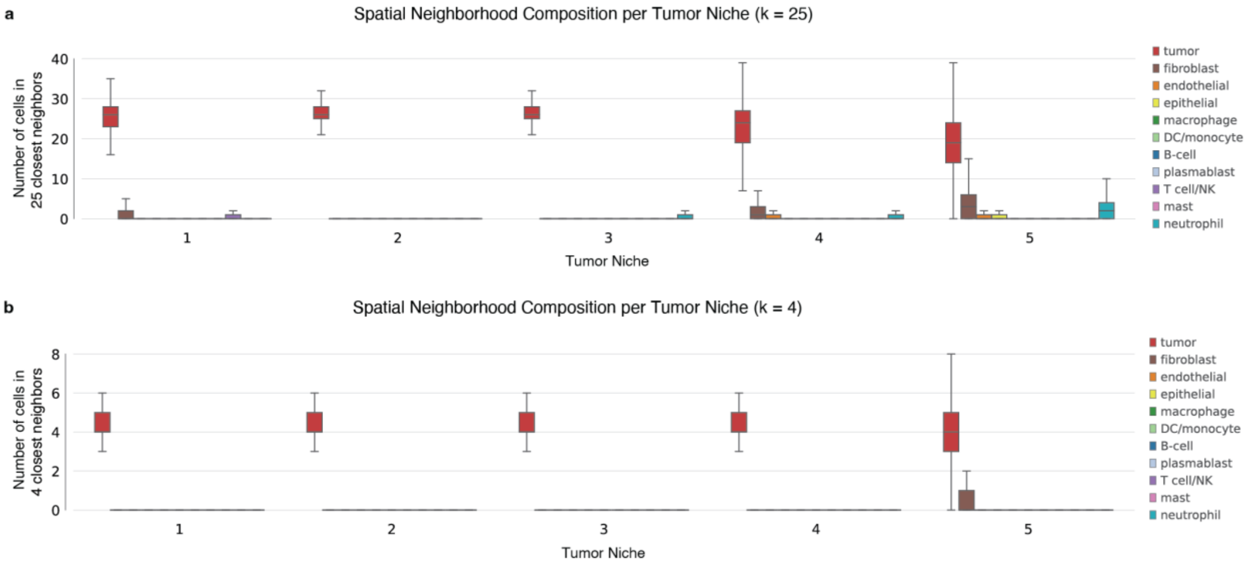

Supplementary Fig. 27 | Tumor niches neighborhood composition. a,**b**, Cell type composition in the spatial neighborhood of cells in tumor niches (niche 1: n = 81,577 cells, niche 2: n = 59,263 cells, niche 3: n = 38,937 cells, niche 4: n = 34,920 cells, niche 5: n = 10,820 cells), using a symmetric k-nearest neighbors graph with 25 neighbors (**a**) and 4 neighbors (**b**). The spatial segregation of tumor cells is evident, as tumor niches predominantly consist of cells surrounded exclusively by other tumor cells, resulting in the identification of pure tumor niches. Boxplot elements are defined as: center line, median; box limits, upper and lower quartiles; whiskers, 1.5x interquartile range.

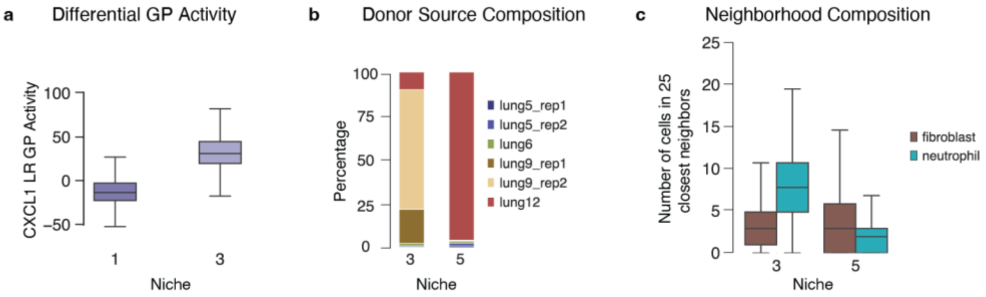

Supplementary Fig. 28 | Tumor niches interacting with neutrophils. a, CXCL1 ligand-receptor program activity distribution in cells from niche 1 (n = 52,076) and niche 3 (n = 29,257) in donor 9. Boxplot elements are defined as: center line, median; box limits, upper and lower quartiles; whiskers, 1.5x interquartile range. **b**, Donor composition of niches 3 and 5. **c**, Neighborhood composition in tumor niches 3 (n = 4,253 cells) and 5 (n = 13,466 cells) in donor 12. Each boxplot represents the distribution of neighboring cells of a given cell type among the 25 physically closest cells for each cell in the niche. Boxplot elements are defined as in **a**. For clarity, only cell types composing on average more than 5% and less than 60% of the neighborhood of any niche are shown. LR: ligand-receptor. GP: gene program.

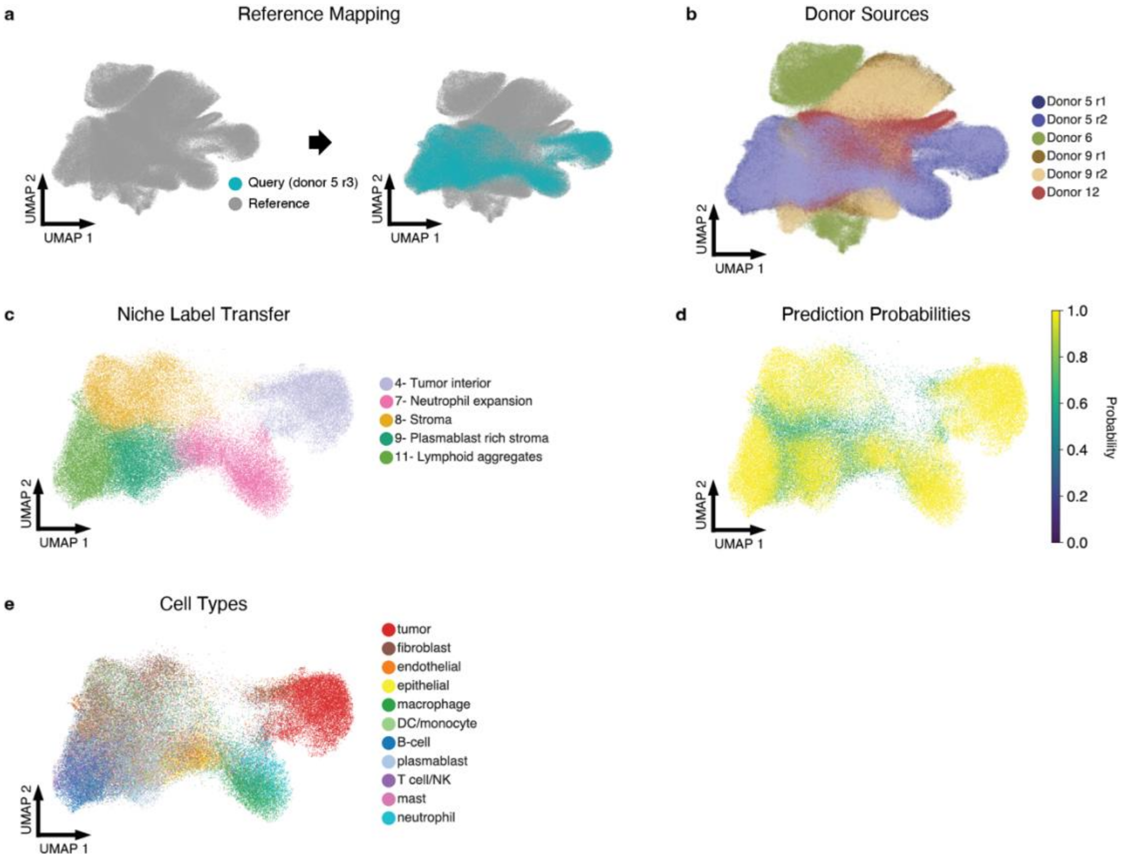

Supplementary Fig. 29 | NicheCompass integrates unseen biological replicates with shared biology into a reference atlas. **a**,**b**, UMAP representation of reference and query cells in NicheCompass embedding space, obtained by mapping query cells onto the reference with fine-tuning, colored by mapping entity (**a**) and sample donor and replicate (**b**). **c**,**d**,**e**, UMAP representation of query cells in the NicheCompass embedding space, colored by niche label (**c**) as predicted by a k-nearest neighbors classifier trained on the reference, prediction probability of the classifier (**d**), and pre-annotated cell type (**e**).

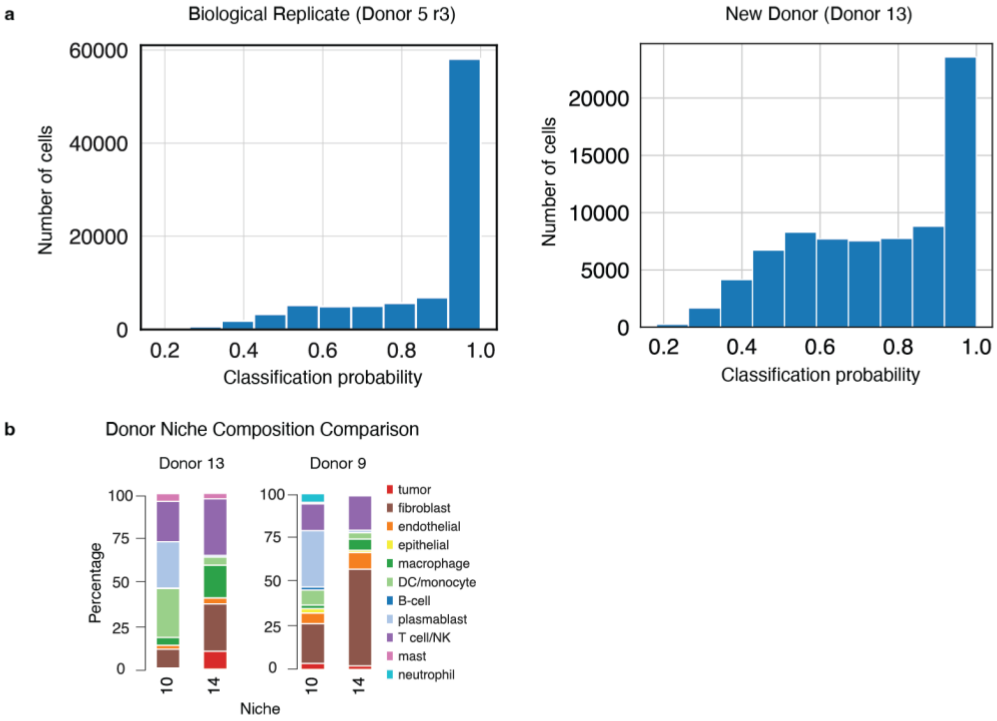

Supplementary Fig. 30 | Label transfer from reference to query distinguishes seen from unseen niches. a, Niche classification probability distribution obtained from a k-nearest neighbors classifier trained on the reference atlas and applied to an unseen biological replicate (left) and an unseen donor (right). **b**, Bar plots representing the cellular composition of the infiltrating stromal niches 10 and 14, which are shared between the new donor 13 (left) and the reference donor 9 (right).

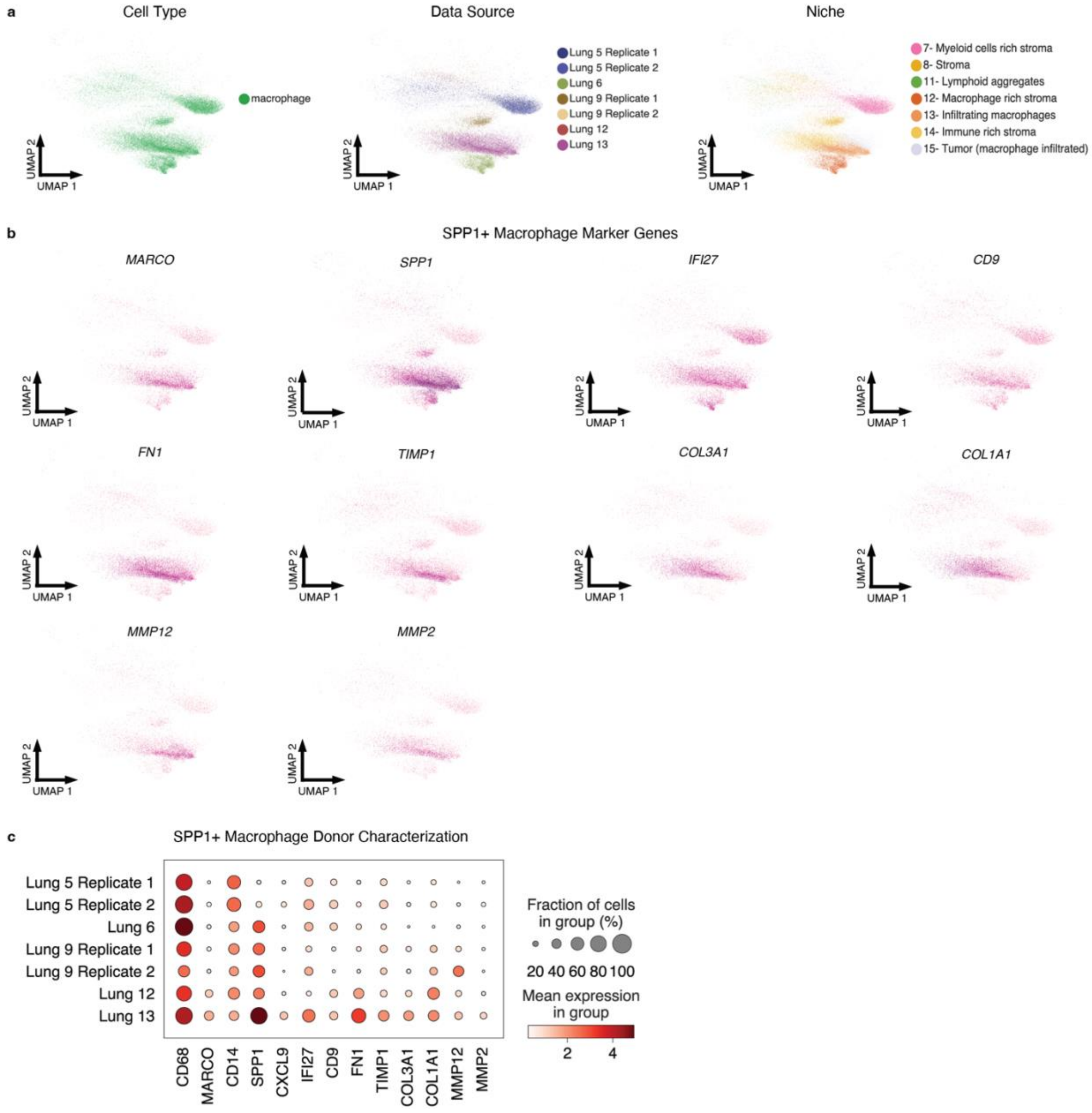

Supplementary Fig. 31 | Characterization of infiltrating macrophages in donor 13 as SPP1+ macrophages. a, UMAP highlighting the macrophages in the integrated NicheCompass embedding space, colored by cell type, data source and identified niche. **b**, Expression levels of genes characteristic of SPP1+ macrophages and its profibrotic phenotype[16-19], significantly overexpressed in donor 13 macrophages compared to macrophages from other donors. **c**, Dot plot illustrating the mean expression levels of genes from panel **b** and general monocyte-macrophage marker genes across macrophages from different donors. Color is proportional to mean expression in a sample, while the dot size represents the percentage of cells expressing that gene.

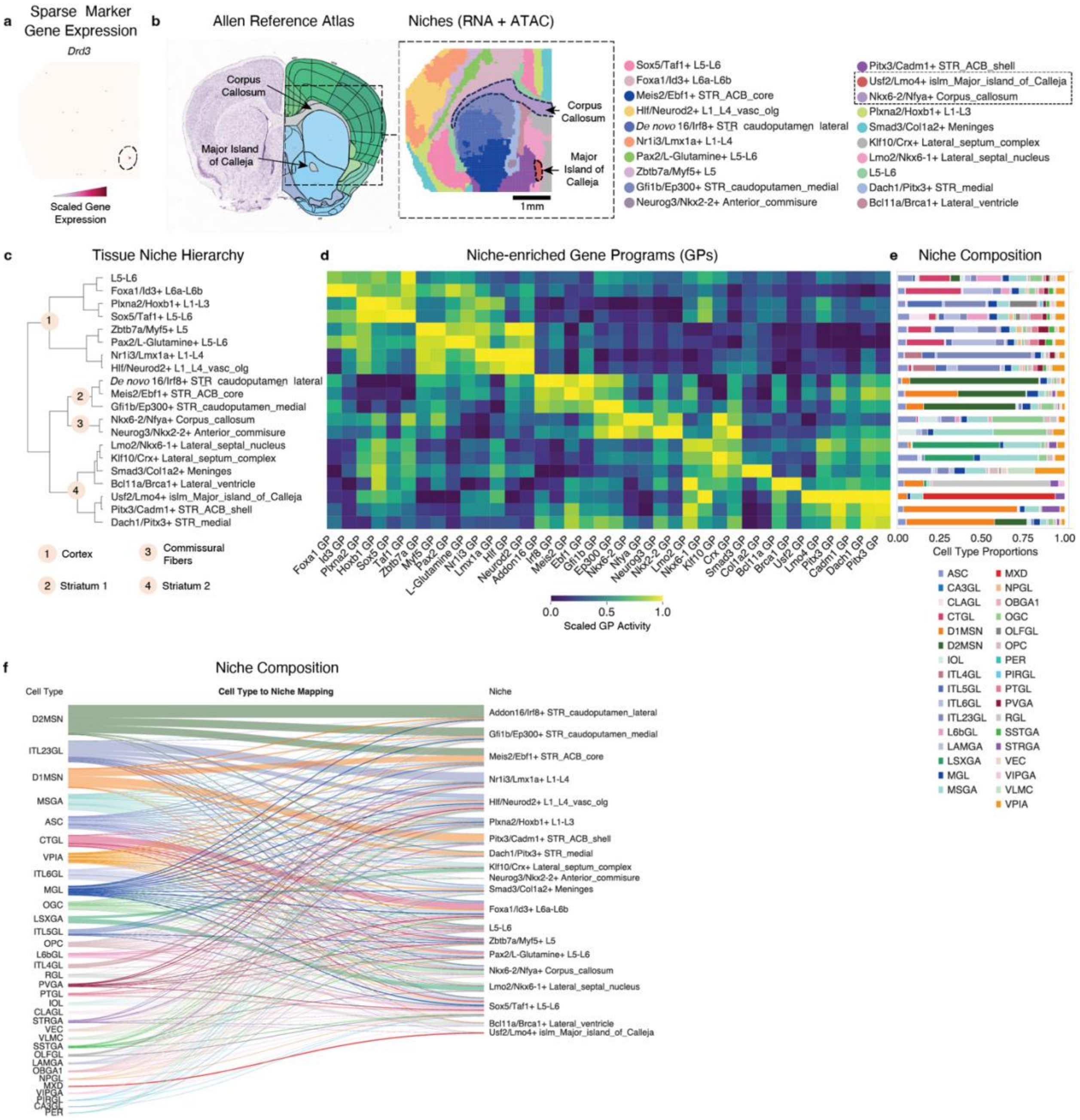

Supplementary Fig. 32 | **Spatial ATAC-RNA seq mouse brain tissue niche organization. a**, Sparse gene expression of the dopamine D3 receptor *Drd3* gene, a marker gene of the Major Island of Calleja[20]. **b**, A coronal mouse brain image from the Allen Brain Atlas showing the Corpus Callosum and Major Island of Calleja; next to it the mouse brain tissue sequenced with the Spatial ATAC-RNA-seq technology[21], highlighting corresponding niches obtained with NicheCompass. The nomenclature of niches is derived from two characterizing programs and niche localization (names in labels refer to upregulated programs). STR: striatum; ACB: nucleus accumbens. **c**, A dendrogram computed based on average program activities of cells in the identified niches, showing a functional niche hierarchy of higher-order functional components. **d**, A heatmap containing for each niche from **c** the normalized activity of two characterizing programs. A gradient along the clustering obtained from **c** is visible. **e**, Cell type proportions for each niche from **c** (based on Zhang et al.[21] cellular annotations for each pixel). **f**, Mapping of the original cell type annotations to the clusters/niches obtained from clustering the NicheCompass embedding space. GP: gene program.

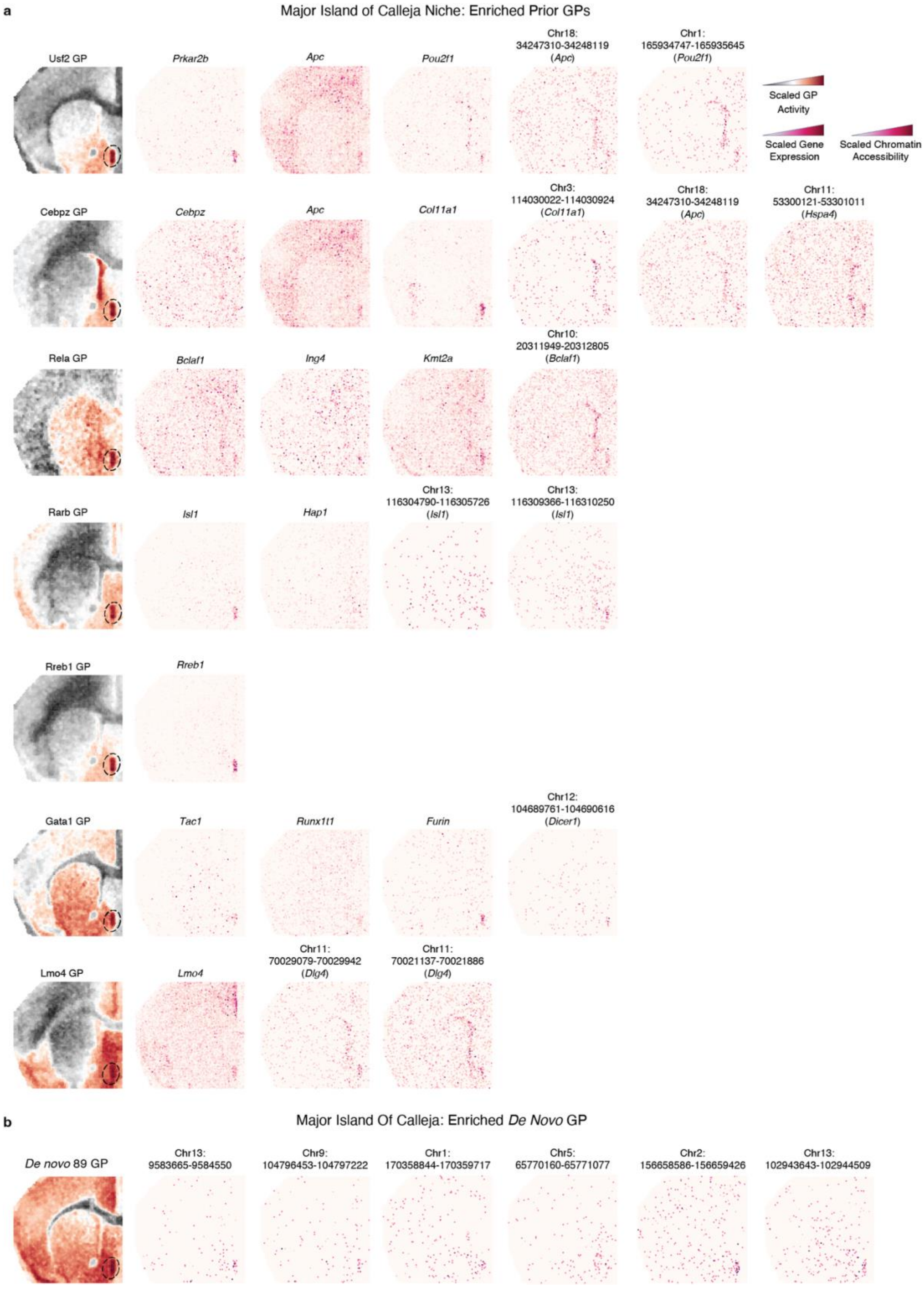

Supplementary Fig. 33 | Enriched programs in the Major Island of Calleja niche. a, The program activity, gene expression of important program genes and peak counts of important program peaks of enriched prior programs in the Major Island of Calleja niche. **b**, Same as **a** but showing an enriched *de novo* program. GP: gene program.

a

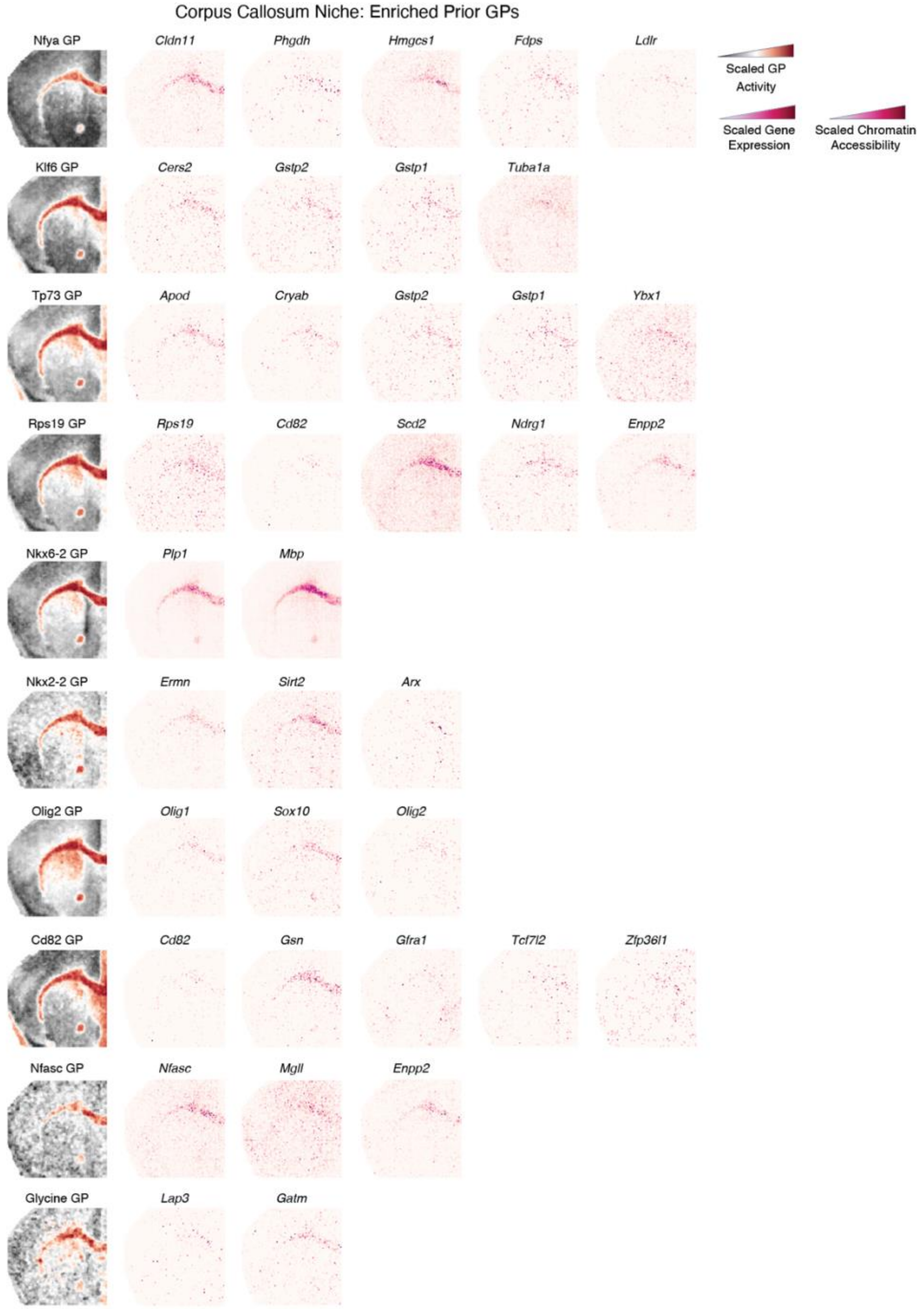

b

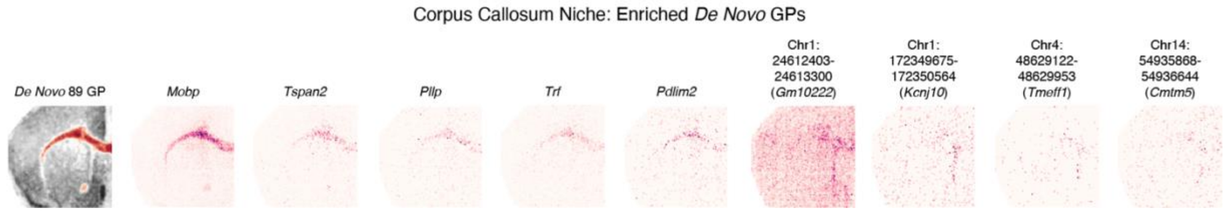

Supplementary Fig. 34 | **Enriched programs in the Corpus Callosum niche. a**, The program activity, gene expression of important program genes and peak counts of important program peaks of enriched prior programs in the Corpus Callosum niche. **b**, Same as **a** but showing an enriched *de novo* program. GP: gene program.

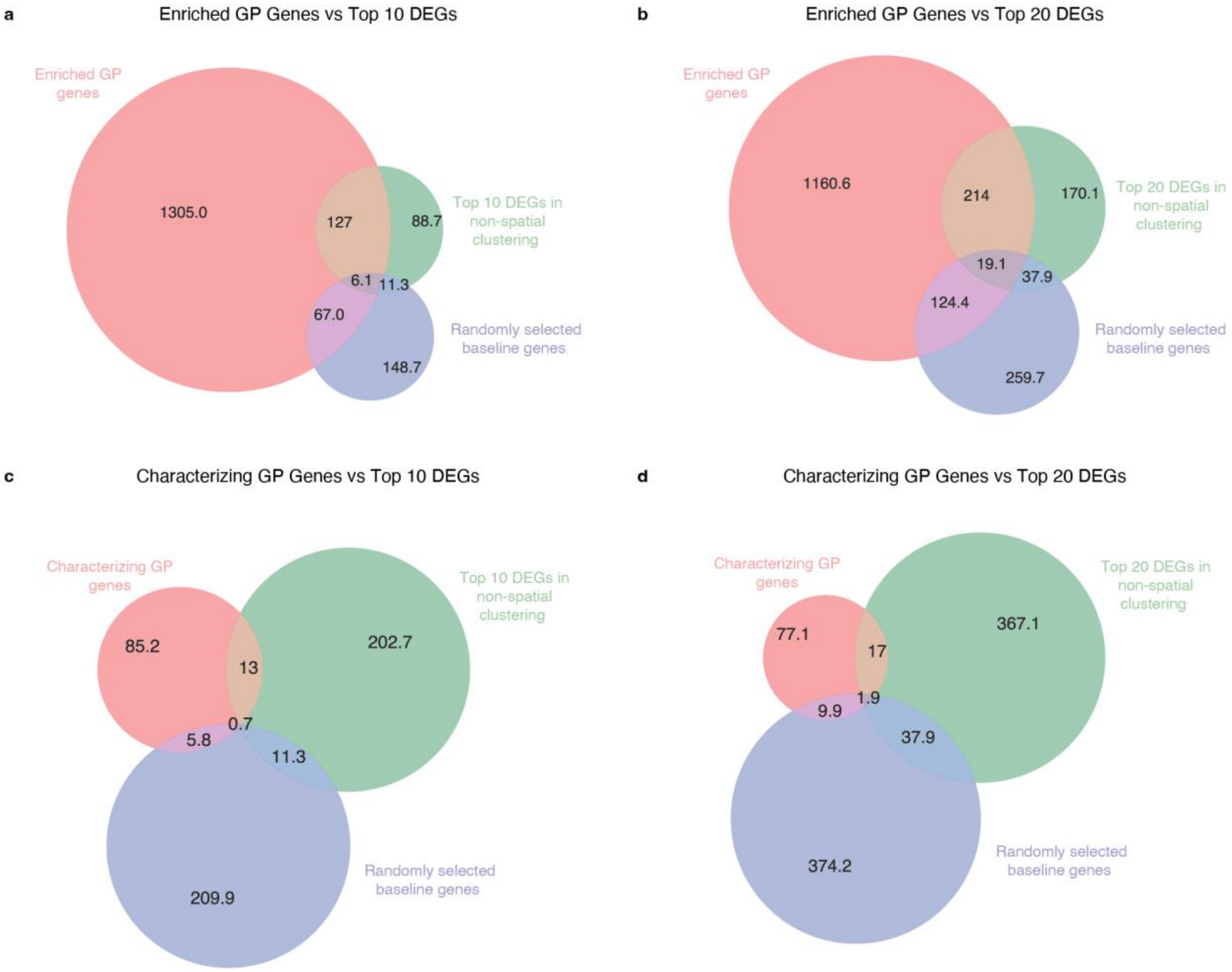

Supplementary Fig. 35 | Overlap between program genes and differentially expressed genes from non-spatial analysis in the seqFISH mouse organogenesis analysis. Venn diagrams illustrate the overlap between program genes with an importance score greater than 0.05 and top differentially expressed genes (DEGs) after clustering the PCA-reduced gene expression space. **a**,**b**, Overlap between enriched program genes and top 10 DEGs (**a**) or top 20 DEGs (**b**). **c**,**d**, Overlap between characterizing program genes and top 10 DEGs (**c**) or top 20 DEGs (**d**). Additionally included is a baseline of randomly selected genes across n = 10 random seeds, matching in number the top DEGs. Number labels in the Venn diagram indicate averages across seeds. While the overlap of randomly selected genes with program genes is significantly lower than the overlap of DEGs with program genes, many program genes are not retrieved with non-spatial analysis. GP: gene program.

Supplementary Tables

	CT 1	CT 2	CT 3	CT 4	CT 5	CT 6	CT 7	CT 8
Niche 1	70%	10%	10%	10%	0	0	0	0
Niche 2	10%	70%	10%	10%	0	0	0	0
Niche 3	10%	10%	50%	10%	10%	10%	0	0
Niche 4	10%	10%	10%	50%	10%	10%	0	0
Niche 5	0	0	0	0	100%	0	0	0
Niche 6	0	0	0	0	0	100%	0	0
Niche 7	12.5%	12.5%	12.5%	12.5%	12.5%	12.5%	12.5%	12.5%
Niche 8	0	0	0	0	25%	25%	25%	25%

Supplementary Table 1 | Data simulation niche composition. Cell type composition of each of the 8 niches in the simulated tissue. CT: Cell Type.

Supplementary Notes

Supplementary Note 1: Description of prior program categories

Prior programs are classified based on the interaction mechanism of their associated pathways. Cell-cell communication programs represent intercellular interactions and include ligand-receptor programs (Supplementary Fig. 1a) and metabolite-sensor programs (Supplementary Fig. 1b). Transcriptional regulation programs represent intracellular interactions, comprising regulons of transcription factors and target genes (Supplementary Fig. 1c). Combined interaction programs encompass linked inter- and intracellular interactions and include ligands, receptors, and downstream target genes (Supplementary Fig. 1d).

Supplementary Note 2: NicheCompass applications

NicheCompass facilitates a comprehensive workflow for analyzing spatial omics data with the following capabilities:

1. Integration of tissue samples to build comprehensible spatial atlases**:**

   NicheCompass integrates disparate tissue samples without requiring prior coordinate alignment and enables the construction of large-scale, comprehensible spatial atlases. Effective integration is achieved using sample-specific neighborhood subgraphs and covariate embeddings to mitigate confounding effects. Its scalability is powered by a memory-efficient PyTorch Geometric[22] framework, incorporating sparse data representations, graph mini-batch data loaders, and neighbor sampling during training. As a result, NicheCompass can seamlessly process datasets with millions of cells or spots. Comprehensibility is obtained via interpretable cell embeddings that enable statistical testing to compare molecular mechanisms and spatial biomarkers across donor samples or disease states. Non-integrating samples can be examined via differential program activity.

2. Biologically informed niche identification**:**

   NicheCompass enables highly performant, biologically informed niche identification via clustering of its learned embeddings. The resulting clusters represent niches that are distinctly defined based on the common cellular processes of its cells, as reflected in shared activity patterns of programs. Varying the cluster resolution can uncover interesting niche hierarchies, reflecting different levels of tissue organization.

3. Detailed niche annotation and characterization**:**

   Cellular program activities are used to annotate and characterize niches. Differential testing of these activities can elucidate niche-specific cellular processes and highlight contrasts between niches. Our interoperable Python package (https://github.com/Lotfollahi-lab/nichecompass), built with a backbone supporting the established scverse ecosystem[23], simplifies further functional niche characterization and frictionless analyses at spatial and single-cell level. For instance, niches can be additionally characterized through cell type composition and neighborhood enrichment of specific cell types.

4. Inference of cellular signaling processes:

   The learned activity of cell-cell communication and combined interaction programs enables inference of cellular signaling processes (Methods). With cell type labels, NicheCompass can identify cell-type-specific interaction patterns within niches.

5. Iterative integration via spatial reference mapping:

   NicheCompass supports transfer learning to iteratively integrate query samples with pre-built spatial reference atlases[24]. This is particularly useful for large-scale atlas building efforts where training data is not available simultaneously. For example, a reference atlas of healthy samples can be used to map diseased samples, enabling niche annotation transfer. Newly discovered niches absent in the reference can be characterized based on differential program activity.

Supplementary Note 3: Additional characterization of gut and brain niches

We identified additional characterizing programs in gut and brain niches. In the Ventral Gut niche, we observed enriched activity of the Indian Hedgehog (Ihh) ligand-receptor program (Extended Data Fig. 1), suggesting signaling between the Ihh protein and the multipass membrane protein Smo. This pathway is crucial for healthy gut development during mouse organogenesis as mutations affecting it lead to gastrointestinal malformations[25-27]. In the Dorsal Gut niche, we identified significant upregulation of the Pdgfc combined interaction program (Extended Data Fig. 1), which, like the Cthrc1 combined interaction program, was specific to the notochord niche. The *Pdgfc* gene, encoding its ligand, is known to be expressed in the notochord[28].

Concerning the brain niches, we found enriched activity of the Gdf10 combined interaction program in the Hindbrain niche (Extended Data Fig. 1), primarily driven by the ligand-encoding gene *Gdf10*. *Gdf10* is implicated in Bergmann glial cell development under Sonic Hedgehog (Shh) regulation in the cerebellum, part of the hindbrain[29]. Aligned with this finding, the Shh combined interaction program, a key signaling pathway during brain development and morphogenesis[30-31] was active across all brain niches with enriched activity in the neighboring Floor Plate niche where the *Shh* gene was also highly expressed (Extended Data Fig. 1). In the Midbrain niche, we observed enriched activity of the Efna2 combined interaction program (Extended Data Fig. 1), driven by the *Efna2* gene. This gene encodes the ephrin A2 protein, crucial for region-specific apoptosis during early brain development[32]. In the Forebrain niche, there was upregulation of the Psck1n ligand-receptor program, which highly correlated with *Psck1n* gene expression (Extended Data Fig. 1).

Supplementary Note 4: Communication potential scores and communication strengths

Program activities quantify how strongly present pathways are within cells/spots, considering their microenvironment (neighborhood). High activities can therefore result from high counts of neighborhood component omics features in a cell's/spot's neighborhood or high counts of self-component omics features in a cell/spot itself, where the highest activity is expected if both coincide. While these activities are useful to quantify the presence of spatial pathways, they are not directly suited for inferring cell-cell communication events between specific interaction partners. To address this, we propose source and target communication potential scores, representing the source- and target-specific pathway activities of cells/spots. Communication potential scores build on inferred program activities of cell-cell communication or combined interaction programs and serve as robust intermediate measures for

the inference of cell-cell communication events. Communication strengths in turn build on communication potential scores to quantify the likelihood for cell-cell communication events between specific interaction partners (Methods).

Supplementary Note 5: SlideSeqV2 mouse hippocampus extended benchmarking

Other benchmarked spatial methods also identified non-contiguous niches that did not meet our niche definition. These clusters, labeled as "Artifacts", primarily captured inhibitory neuron populations (Fig. 3c and Supplementary Fig. 8b; *Gad2* expression in Allen ISH atlas). All methods also faced challenges in accurately recovering the L6b layer, which is much thinner than the identified laminar regions. BANKSY[5] struggled with thalamic nuclei differentiation and identification of the fasciola cinerea niche, which based on marker expression[9] was clearly delineated from its neighboring niches (Supplementary Fig. 8c). GraphST[6] failed to resolve cortical laminar structure, and CellCharter[7] and DeepLinc[8] smeared the pyramidal layer in CA1-3 beyond its cytoarchitectural boundaries. Hierarchical clustering showed slight differences among methods compared to the anatomical hierarchy, albeit all methods recovered reasonable structures (Fig. 3c and Supplementary Fig. 8b).

In addition to spatial methods, we also applied two non-spatial deep generative methods, scVI[33] and expiMap[34], to assess whether they capture niches based on tissue anatomy in their embeddings. As expected, the resulting embeddings were more influenced by intrinsic cell type variation, leading to spatially scattered clusters. This reflects that cells of the same cell type are in different parts of the tissue and highlights the need for spatial methods to adequately capture spatially consistent niches of heterogeneous cell. Nonetheless, these methods also partially recovered tissue anatomy independent of cell type, with some spatially adjacent cells of different cell types located close to each other in embedding space (Supplementary Fig. 8d,e). This emphasizes that some spatial effects are already evident in single-cell transcriptomes (without considering the microenvironment).

Moreover, we also performed clustering on the spatial coordinate feature space to investigate whether cell positioning alone would lead to reasonable niches (Supplementary Fig. 8f). As expected, this resulted in spatially consistent niches, yet these did not correspond to anatomical structures, highlighting the importance of incorporating molecular information.

Regarding benchmarking on the 25% subsample, NicheCompass maintained superior spatial consistency compared to BANKSY, GraphST, CellCharter, and DeepLinc. STACI[13] failed to separate the CA1 niche from the Stratum niche, reflected in a low niche coherence score (Supplementary Fig. 9b).

Supplementary Note 6: Metrics

Building on prior work[1,35-37], we introduce metrics to evaluate cell embeddings in the context of niche identification, applicable to both single-sample and sample integration scenarios. These metrics are categorized into three groups, spatial consistency (CAS, CLISIS, MLAMI, GCS), niche coherence (NASW, CNMI), and batch correction (BLISI, PCR), and reflect essential criteria to identify spatially contiguous, biologically meaningful niches. First, cell embeddings should capture tissue patterns of spatially co-occurring cell types and the spatial organization of tissues at various resolutions. This enables the identification of fine-grained substructures that merge into coarser niches, reflecting hierarchical tissue organization. We therefore include two metrics to measure global spatial conservation of the tissue architecture: Cell Type Affinity Similarity (CAS) and Maximum Leiden Adjusted Mutual Information (MLAMI) (Supplementary Methods). Second, proximal cells in physical space should also be proximal in embedding space to ensure niches are composed of neighboring cells. We measure this local dimension of spatial consistency through two additional metrics: Cell Type Local Inverse Simpson's Index (CLISIS) and Graph Connectivity Similarity (GCS) (Supplementary Methods). Third, niches should reflect biologically coherent cell groupings rather than random spatial associations, which is the case when molecular features are not considered (Supplementary Fig. 8f). To ensure this, we measure how compact niches are within themselves while being distinct from neighboring niches, using the Niche Average Silhouette Width (NASW), and how pure niches are in terms of cell type composition, using the Cell Type Normalized Mutual Information (CNMI; Supplementary Methods). We combine these two metrics into the niche coherence score.

To compare methods holistically, we further aggregate all metrics into an overall score through normalization and equal weighting of the spatial consistency, niche coherence, and batch correction (when integrating samples) categories. This ensures that niches are both spatially and biologically coherent. Users may adjust the weighting to emphasize specific criteria based on their biological questions.

Supplementary Note 7: Extended benchmarking on simulated data

We computed all six metrics for spatial consistency and niche coherence, along with four additional metrics from the SDMBench[38], to measure the correspondence between identified and ground truth niches across n = 8 model runs with varying neighborhood sizes. This quantitative evaluation aligned with our qualitative observations, showing high consistency between our metrics and the supervised SDMBench metrics (Extended Data Fig. 3e). Next, we compared NicheCompass' program inference with two alternative workflows based on BANKSY-inferred niches. In the first workflow, we performed niche-level gene set enrichment analysis[39] of program target genes (Methods). In the second workflow, we conducted niche-level cell-cell interaction inference using LIANA[40] (Methods). Both workflows failed to retrieve ground truth programs as accurately as NicheCompass (Extended Data Fig. 3f), highlighting the advantage of incorporating prior programs during model training over relying on post-hoc inference.

Supplementary Note 8: Ablation studies and guidance on hyperparameter selection

We performed ablation studies on simulated and real data to evaluate key design choices and hyperparameters, including: (1) weighting of edge and gene expression reconstruction losses, (2) regularization of omics decoder weights for prior and *de novo* programs, (3) encoder architecture (GCNconv vs GATv2conv layers), (4) number of neighbors in the k-NN graph, (5) number of *de novo* programs, (6) program pruning, and (7) a version of NicheCompass without prior programs, i.e. fully connected omics decoders (Methods).

We first describe the results on simulated data (Supplementary Fig. 10-11). Regarding the loss, we observed that finding a balance between gene expression and edge reconstruction is a key element for good niche identification (NID) and gene program recovery (GPR) performance, while regularization of *de novo* program weights is essential for GPR and especially the correct retrieval of important *de novo* genes. In fact, most other hyperparameters had negligible effects on the identification of *de novo* program genes in the absence of regularization of *de novo* program weights; exceptions are (1) the number of *de novo* programs, where a too high number can lead to an increase in false positives and to unwanted dissemination of interaction signals across multiple programs (Supplementary Fig. 11c), and (2) the neighborhood size, which should not be too large compared to the expected range of interactions as different signals can get mixed within individual *de novo* programs. The loss weights have been specified in the NicheCompass package accordingly and, in our experience, do not need to be changed by the user. In terms of encoder layer, the NicheCompass Light variation with a GCN layer could achieve better NID on this dataset while the GATv2 layer led to better GPR. With respect to size of the k-NN graph, a smaller number of neighbors was more efficient in NID and *de novo* program detection while a larger number of neighbors facilitated GPR of prior

programs, likely due to the sparsity of interactions in our simulated data and the constrained nature of prior programs which benefit from signal enhancements through larger neighborhood sizes (as opposed to *de novo* programs which are not constrained and can suffer from mixed signals with larger neighborhood sizes). Here, we recommend users to specify a neighborhood size based on the expected range of interactions in the tissue. Empirically, we observed a neighborhood size between 4 and 12 to work well. Importantly, the inclusion of *de novo* programs was crucial for GPR with a default number of 100 *de novo* programs leading to a good balance between NID and GPR. We recommend users to keep this default unless prior programs are customized. Moreover, program pruning slightly improved GPR and NID while reducing the embedding size of the model; we therefore recommend users to use the default setting of weak program pruning. Finally, we performed an ablation on a version of NicheCompass without prior programs. On this dataset, we observed good NID performance, but this came at the cost of program gene recovery preventing an inherent niche characterization. Based on this finding and the impact analysis of different prior program sets presented in Supplementary Fig. 6, we recommend that users define prior programs solely based on the biology that they are interested in (as opposed to including as many prior programs as possible).

In our ablation study on real data, we found similar optimal configurations for most hyperparameters compared to the simulated data, showing that hyperparameters are quite robust across specific spatial patterns and gene expression sensitivity (Supplementary Fig. 13). Specifically, a balance between the gene expression and edge reconstruction losses was crucial for good NID performance and program gene weight regularization led to further improvements. Lower neighborhood sizes were also advantageous for NID in real data; in contrast, with real data, the GATv2 layer performed better in terms of NID, which was also the case in our benchmarking study on most datasets (Supplementary Figs. 16-19 and 21-23), likely due to the increased complexity of real data compared to simulated data requiring a more expressive model architecture. This performance difference was even more pronounced with spot-resolution data (Supplementary Fig. 14f). We therefore recommend users to use a GATv2 encoder layer (as opposed to NicheCompass Light) unless performance is a bottleneck or niche characterization is not a priority and the data has single-cell resolution. Additionally, like with simulated data, program pruning could slightly boost NID performance while leading to lower embedding sizes, albeit stronger program pruning was necessary. Finally, the use of prior programs significantly improved NID compared to a scenario without prior programs. Based on these findings, we offer model training recommendations in our package user guide (https://nichecompass.readthedocs.io/en/latest/).

Supplementary Note 9: Spot resolution benchmarking

While niche identification remained robust with spot-level resolution, performance was slightly lower, particularly in correctly identifying niche peripheries, and program recovery was significantly worse (Supplementary Fig. 14c,d). We also compared NicheCompass with other methods[5-7] on a spot-resolution Stereo-Seq mouse embryo dataset[12] (Supplementary Fig. 14e) with ground truth niche labels. NicheCompass was among the top-performing methods, as assessed by both our and SDMBench[38] metrics (Supplementary Fig. 14f).

Supplementary Note 10: Extended NanoString CosMx human NSCLC benchmarking

We also tested method variants including GraphST without prior alignment through PASTE and a version of NicheCompass without the field of view as a covariate. While NicheCompass remained the only method to successfully remove field of view effects, excluding the field of view covariate caused integration failure, highlighting the importance of covariate embeddings.

Supplementary Note 11: Scalability and speed

While NicheCompass seamlessly scaled to large datasets, other methods (except for CellCharter and BANKSY) were limited by loading the full graph into memory (instead of mini-batch training). This led to memory overflow when loading big datasets on our 40GB GPU (Supplementary Fig. 24a,b). In smaller data regimes, other methods exhibited faster runtimes, mainly due to the graph attention mechanism employed by our model. In fact, the NicheCompass Light variation of our model, which uses convolutional layers, showed competitive runtimes in the small data regime (Supplementary Fig. 24a,b), and performed comparably to NicheCompass regarding the overall score (Supplementary Figs. 16-19 and 21-23).

Notably, in our benchmarking experiments, NicheCompass' runtime was significantly impacted by the number of genes available in the dataset (Supplementary Fig. 24a,b). This is due to the following reasons: (1) Only programs are included for which genes are present in the dataset; hence, a higher number of genes usually implies a higher number of programs, which corresponds to a bigger model embedding size. (2) An increasing number of genes not only increases the number of neurons in the fully connected output layer of the decoder but also the number of connections between the hidden layer and output layer as programs will consist of more genes. (3) While the previous two points increase the number of model parameters (Supplementary Fig. 24c,d), hence leading to slower run times per se, we have optimized the batch size for each model run to use our available GPU memory. As a result, we were able to use bigger batch sizes for datasets with fewer genes which led to a significant speed up.

Supplementary Note 12: Spatial ATAC-RNA-seq mouse brain niche annotation & characterization

To annotate the niches identified by NicheCompass we determined two characterizing programs for each niche and used these in conjunction with the Allen Brain Atlas[4] to obtain functional and anatomical annotations. Subsequently, we built a niche hierarchy to decipher the tissue's global spatial organization (Supplementary Fig. 32c). We observed that niches mainly clustered into higher-order anatomical structures such as the cortical layers constituting a Cortex cluster (1) and spatially adjacent niches of the striatum grouping into two Striatum clusters (2,4). Due to their molecular similarities, the Corpus Callosum and Anterior Commissure niches formed a commissural fiber cluster (3), despite being in distant tissue regions. In addition, the characterizing programs of each niche again showed distinctive signatures, consistent with the identified hierarchy (Supplementary Fig. 32d), and niches adjacent in the hierarchy showed similar cell type composition (Supplementary Fig. 32e).

The Major Island of Calleja, containing dopamine D3 receptor-expressing granule cells and expressing *Gad2* and *Nos1*[41,42], lacks detailed molecular characterization. Enrichment analysis revealed interesting transcriptional regulation programs with multimodal footprints (Supplementary Fig. 33a), including the Usf2 program (driven by target genes *Prkar2b*, *Apc*, *Pou2f1* and associated peaks), which regulates glycolytic glucose metabolism[43-46], and the Rreb1 program, marking a GABAergic spiny projection neuron subpopulation[47]. An interesting *de novo* program with correlated peaks was also identified (Supplementary Fig. 33b).

In the Corpus Callosum niche, we found enriched programs linked to oligodendrocyte functions (Supplementary Fig. 34). These included transcriptional regulation programs like Klf6, essential for CNS myelination[48], ligand-receptor programs such as Cd82, involved in oligodendrocyte differentiation[49,50], and metabolite-sensor programs like the Glycine program driven by *Gatm*, involved in the synthesis of creatine in oligodendrocytes[51]. A *de novo* program highlighted genes (*Mobp*, *Tspan2*, *Pllp*, *Trf*, *Pdlim2*) associated with myelinating oligodendrocytes[52-55].

Supplementary Methods

NicheCompass model: additional information

Encoder. Graph attention layers are defined node-wise as:

$$\boldsymbol{\mu}_i = \frac{1}{N_{\text{head}}} \sum_{n=1}^{N_{\text{head}}} \left[\alpha_{i,i}^{(n)} \mathbf{W}^{(\text{enc\_}\mu\text{\_}s)} \mathbf{h}_i + \sum_{j \in \mathcal{N}(i)} \alpha_{i,j}^{(n)} \mathbf{W}^{(\text{enc\_}\mu\text{\_}t)} \mathbf{h}_j \right]$$

$$log(\boldsymbol{\sigma}_i) = \frac{1}{N_{\text{head}}} \sum_{n=1}^{N_{\text{head}}} \left[\beta_{i,i}^{(n)} \mathbf{W}^{(\text{enc\_}log(\boldsymbol{\sigma})\text{\_}s)} \mathbf{h}_i + \sum_{j \in \mathcal{N}(i)} \beta_{i,j}^{(n)} \mathbf{W}^{(\text{enc\_}log(\boldsymbol{\sigma})\text{\_}t)} \mathbf{h}_j \right]$$

where the attention coefficients of attention head n, $\alpha_{i,j}^{(n)}$ and $\beta_{i,j}^{(n)}$, are computed as:

$$\alpha_{i,j}^{(n)} = \frac{\exp\left(\mathbf{a}^{(\text{n,enc\_}\mu)^T} \text{LeakyReLU}\left(\mathbf{W}^{(\text{enc\_}\mu\text{\_}s)} \mathbf{h}_i + \mathbf{W}^{(\text{enc\_}\mu\text{\_}t)} \mathbf{h}_j\right)\right)}{\sum_{m \in \mathcal{N}(i) \cup \{i\}} \exp\left(\mathbf{a}^{(\text{n,enc\_}\mu)^T} \text{LeakyReLU}(\mathbf{W}^{(\text{enc\_}\mu\text{\_}s)} \mathbf{h}_i + \mathbf{W}^{(\text{enc\_}\mu\text{\_}t)} \mathbf{h}_m)\right)}$$

$$\beta_{i,j}^{(n)} = \frac{\exp\left(\mathbf{a}^{(\text{n,enc\_}log(\boldsymbol{\sigma}))} \text{LeakyReLU}\left(\mathbf{W}^{(\text{enc\_}log(\boldsymbol{\sigma})\text{\_}s)} \mathbf{h}_i + \mathbf{W}^{(\text{enc\_}log(\boldsymbol{\sigma})\text{\_}t)} \mathbf{h}_j\right)\right)}{\sum_{m \in \mathcal{N}(i) \cup \{i\}} \exp\left(\mathbf{a}^{(\text{n,enc\_}log(\boldsymbol{\sigma}))^T} \text{LeakyReLU}(\mathbf{W}^{(\text{enc\_}log(\boldsymbol{\sigma})\text{\_}s)} \mathbf{h}_i + \mathbf{W}^{(\text{enc\_}log(\boldsymbol{\sigma})\text{\_}t)} \mathbf{h}_m)\right)}$$

$\mathbf{W}^{(\text{enc\_}\mu\text{\_}s)}, \mathbf{W}^{(\text{enc\_}\mu\text{\_}t)}, \mathbf{W}^{(\text{enc\_}log(\boldsymbol{\sigma})\text{\_}s)}, \mathbf{W}^{(\text{enc\_}log(\boldsymbol{\sigma})\text{\_}t)} \in \mathbb{R}^{N_{\text{gp}} \times N_{\text{hid}}}$ and $\mathbf{a}^{(\text{n,enc\_}\mu)}, \mathbf{a}^{(\text{n,enc\_}log(\boldsymbol{\sigma}))} \in \mathbb{R}^{N_{\text{gp}}}$ are learnable weights (biases omitted for simplicity).

Graph convolutional layers in NicheCompass Light are defined node-wise as:

$$\boldsymbol{\mu}_i = \mathbf{W}^{(\text{enc\_}\mu)} \sum_{j \in \mathcal{N}(i) \cup \{i\}} \left[\frac{\mathbf{h}_j}{\sqrt{d_j d_i}} \right]$$

$$log(\boldsymbol{\sigma}_i) = \mathbf{W}^{(\text{enc\_}log(\boldsymbol{\sigma}))} \sum_{j \in \mathcal{N}(i) \cup \{i\}} \left[\frac{\mathbf{h}_j}{\sqrt{d_j d_i}} \right]$$

Loss function. The observation-level loss of the modality-specific omics reconstruction losses is:

$$\begin{aligned}
\mathcal{L}_i^{(\text{mod})}\left(\boldsymbol{\phi}_i^{(\text{mod})}, \boldsymbol{\phi}_i'^{(\text{mod})}, \boldsymbol{\theta}^{(\text{mod})}, \boldsymbol{\theta}'^{(\text{mod})}; \mathbf{x}_i^{(\text{mod})}, \mathbf{x}_i'^{(\text{mod})}\right) = \\
\text{NBL}\left(\boldsymbol{\phi}_i^{(\text{mod})}, \boldsymbol{\theta}^{(\text{mod})}; \mathbf{x}_i^{(\text{mod})}\right) + \text{NBL}\left(\boldsymbol{\phi}_i'^{(\text{mod})}, \boldsymbol{\theta}'^{(\text{mod})}; \mathbf{x}_i'^{(\text{mod})}\right) = \\
-\sum_{n=1}^{N_{\text{mod}}} [\, \mathbf{x}_i^{(\text{mod})} \log\left(\frac{\boldsymbol{\phi}_i^{(\text{mod})} + \epsilon}{\boldsymbol{\phi}_i^{(\text{mod})} + \boldsymbol{\theta}^{(\text{mod})} + \epsilon}\right) + \theta^{(\text{mod})} \log\left(\frac{\boldsymbol{\theta}^{(\text{mod})} + \epsilon}{\boldsymbol{\phi}_i^{(\text{mod})} + \boldsymbol{\theta}^{(\text{mod})} + \epsilon}\right) + \\
\log\left(\frac{\Gamma\left(\mathbf{x}_i^{(\text{mod})} + \boldsymbol{\theta}^{(\text{mod})}\right)}{\Gamma\left(\mathbf{x}_i^{(\text{mod})} + 1\right) \Gamma(\boldsymbol{\theta}^{(\text{mod})})}\right)] \\
-\sum_{n=1}^{N_{\text{mod}}} [\, \mathbf{x}_i'^{(\text{mod})} \log\left(\frac{\boldsymbol{\phi}_i'^{(\text{mod})} + \epsilon}{\boldsymbol{\phi}_i'^{(\text{mod})} + \boldsymbol{\theta}'^{(\text{mod})} + \epsilon}\right) + \boldsymbol{\theta}'^{(\text{mod})} \log\left(\frac{\boldsymbol{\theta}'^{(\text{mod})} + \epsilon}{\boldsymbol{\phi}_i'^{(\text{mod})} + \boldsymbol{\theta}'^{(\text{mod})} + \epsilon}\right) + \\
\log\left(\frac{\Gamma\left(\mathbf{x}_i'^{(\text{mod})}\right) + \boldsymbol{\theta}'^{(\text{mod})})}{\Gamma\left(\mathbf{x}_i'^{(\text{mod})} + 1\right) \Gamma(\boldsymbol{\theta}'^{(\text{mod})})}\right)]
\end{aligned}$$

Differential testing of program activities. $p(H_0)$ is computed as:

$$p(H_0) = \mathbb{E}_{p\left(\mathrm{X}^{(1)}|\mathrm{G}^{(1)}=a\right) p\left(\mathrm{X}^{(2)}|\mathrm{G}^{(2)}=b\right)} [p\left(\mathrm{Z}_u^{(1)} > \mathrm{Z}_u^{(2)} | \mathrm{X}^{(1)}, \mathrm{X}^{(2)}\right]\right)$$

where $\mathrm{G}^{(1)}$ and $\mathrm{G}^{(2)}$ denote the independent random variables for group membership and

$$p\left(\mathrm{Z}_u^{(1)} > \mathrm{Z}_u^{(2)} | \mathrm{X}^{(1)}, \mathrm{X}^{(2)}\right) \approx \mathbb{E}_{q_{\mu_1,\sigma_1}\left(\mathrm{Z}_u^{(1)}|\mathrm{X}^{(1)}\right) q_{\mu_2,\sigma_2}\left(\mathrm{Z}_u^{(2)}|\mathrm{X}^{(2)}\right)} \left[I\left(\mathrm{Z}_u^{(1)} > \mathrm{Z}_u^{(2)}\right) \right]$$

Since the approximate posterior $q_{\mu_i,\sigma_i}\left(\mathrm{Z}^{(i)}|\mathrm{X}^{(i)}\right)$ is Gaussian, the expectation can be calculated via:

$$\mathbb{E}_{q_{\mu_1,\sigma_1}\left(\mathrm{Z}_u^{(1)}|\mathrm{X}^{(1)}\right) q_{\mu_2,\sigma_2}\left(\mathrm{Z}_u^{(2)}|\mathrm{X}^{(2)}\right)} \left[I\left(\mathrm{Z}_u^{(1)} > \mathrm{Z}_u^{(2)}\right)] \right] = \frac{1}{2} \text{erfc}\left(\frac{-\mu_{1_u} - \mu_{2_u}}{\sqrt{2\left(\sigma_{1_u}^2 + \sigma_{2_u}^2\right)}} \right)$$

Statistics and reproducibility: additional information

Data simulation. The 1,105 genes were derived as follows: (1) Up to three source and three target genes were sampled from quality-filtered prior programs. (2) An equal number of *de novo* programs with one source and three target genes were created. (3) A subset of 316 programs (171 prior, 145 *de novo*) was selected, comprising the full gene set of 1,105 genes; thereof 543 were present in the NicheCompass prior programs and 562 were not. 50% of these programs were sampled to inject niche-specific ground truth program activity. Ground truth program activity was injected as follows: For each sampled program, we (1) sampled a niche for program upregulation, (2) sampled an increment parameter for upregulation through an additive gene

expression model, (3) sampled a source and target cell type, (4) determined all cells in the sampled niche that were of the sampled target cell type and had cells of the sampled source cell type in their neighborhood (k = 6), and, based on the sampled increment parameter, (5) upregulated the sampled target genes of each program in these cells, and (6) upregulated the sampled source genes in cells in the neighborhood that were of the sampled source cell type.

NicheCompass model configuration. Models were trained with the Adam optimizer (initial learning rate: 10^{-3}), a learning rate scheduler (patience: 4 epochs, reduction factor: 0.1), and early stopping (patience: 8 epochs). Default hyperparameters were used

CellCharter model configuration. Following the tutorial at https://cellcharter.readthedocs.io/en/latest/notebooks/codex_mouse_spleen.html, we used scVI[33] for dimensionality reduction and integration. Default hyperparameters were used to retrieve aggregated cell embeddings.

STACI model configuration. Hidden layer sizes were set to three times the input feature size (approximately equivalent to the the original publication[13]). Training was run for 1000 epochs after which no further improvements were observed. Other hyperparameters were set according to https://github.com/uhlerlab/STACI/blob/master/train_gae_starmap_multisamples.ipynb. The codebase was taken from GitHub (https://github.com/uhlerlab/STACI/blob/master) on 23.11.2023.

GraphST model configuration. We used default hyperparameters for training, following tutorial 1 at https://deepst-tutorials.readthedocs.io/en/latest.

DeepLinc model configuration. We used default hyperparameters for training. The codebase was taken from https://github.com/xryanglab/DeepLinc on 22.05.2023.

BANKSY model configuration. Hyperparameters were set according to the original publication's recommendations. The codebase was taken from https://github.com/prabhakarlab/Banksy on 12.07.2024. Following the vignette from https://github.com/prabhakarlab/Banksy_py/blob/main/slideseqv2_analysis.ipynb, we performed PCA on cell embeddings.

Gene program inference comparison. We trained n = 8 models per method using different seeds and a k-NN graph with k = 6 (matching the ground truth interaction range). Programs were ranked by method-specific scores: NicheCompass by log Bayes factor, BANKSY + GSEA[39] by p-values, and BANKSY + LIANA[40] by magnitude rank. For each niche, the top-ranked programs (equal in number to the ground truth upregulated programs) were selected, and F1 scores were computed against the ground truth. These scores were aggregated across niches to obtain the final F1 score per method. Due to errors in BANKSY + LIANA's rank computation for single-cell-type niches (5 and 6), F1 scores of 0 were assigned to these niches. Final F1 scores were averaged across all training runs.

Model evaluation metrics

General specifications. We construct k-NN graphs using scib.nearest_neighbors.pynndescent()[35] (k = 15 except for CLISIS where k = 50). scIB metrics are computed via the scib-metrics package with default parameters. For clustering, we employ the Leiden algorithm using scanpy.tl.leiden()[56].

CAS. The Cell Type Affinity Similarity (CAS) quantifies the conservation of spatial cell type organization in the embedding space of a model compared to physical (tissue) space. Specifically, it measures how well cell-cell contact maps[1] (cell type enrichment scores in a cell's neighborhood measured across all cell types) are preserved in a model's embedding space and as such quantifies spatial consistency of the embedding space. The CAS is scaled between 0 and 1, with higher values indicating superior cell-cell contact map similarity and hence better preservation of global spatial cell type organization. We first construct a k-NN graph in physical space and count edges between all cell type pairs. We then permute cell type assignments across cells (1,000 times) and repeat edge counting. We derive a physical cell-cell contact map $\mathbf{M}^P \in \mathbb{R}^{N_C \times N_C}$, where N_C is the number of cell types, calculating z-scores for each cell type pair, reflecting the enrichment of connections in the observed data compared to permutations (using squidpy.pl.nhood_enrichment()). The same procedure is applied with a k-NN graph constructed in embedding space producing the latent cell-cell contact map $\mathbf{M}^L \in \mathbb{R}^{N_C \times N_C}$. We then compute $\| \mathbf{M}^L - \mathbf{M}^P \|_F = \sqrt{\sum_{a=1}^{N_C} \sum_{b=1}^{N_C} \left| \mathrm{M}_{a,b}^L - \mathrm{M}_{a,b}^P \right|^2}$ to quantify the distance between $\mathbf{M}^L$ and $\mathbf{M}^P$. We subtract this distance from 1 and perform scaling to obtain the CAS. In integration scenarios, k-NN graphs for each sample are combined as isolated components, while a single embedding graph is constructed from the integrated space.

MLAMI. The Maximum Leiden Adjusted Mutual Info (MLAMI) is an unsupervised metric to evaluate the degree of overlap between clusters of cells in the embedding space of a model and clusters of cells in physical space. The MLAMI quantifies the preservation of global spatial organization and ranges from 0 to 1, with higher values indicating a more accurate preservation of global spatial organization.We construct k-NN graphs based on the physical and embedding spaces and perform clustering across resolutions from 0.1 to 1.0 at 0.45 increments. Similarity of obtained clusters is assessed using the Adjusted Mutual Info (AMI) of all clustering pairs and the maximum score is reported as MLAMI. In integration scenarios physical clusters are computed per sample, while joint clusters are derived from the integrated embedding space. MLAMI is then calculated per sample by comparing embedding clusters with corresponding physical clusters, and the mean MLAMI across all samples is reported.

CLISIS. The Cell Type Local Inverse Simpson's Index Similarity (CLISIS) indicates how well spatial cell type heterogeneity is preserved in the embedding space of a model compared to the physical space. It is based on the Local Inverse Simpson's Index (LISI)[36], and ranges between 0 and 1, with higher values indicating more accurate preservation of the local neighborhood cell type heterogeneity. The cell-level CLISI captures the degree of cell mixing in a local neighborhood around a given cell. For each cell, we compute the ratio of the Cell Type Local Inverse Simpson Indices (CLISI)[35] in the physical and embedding spaces, take the logarithm to get relative local heterogeneity scores[37], normalize by the maximum possible score, and calculate the median absolute value across cells. Subtracting this median from 1 yields the final CLISIS. In integration scenarios, embedding CLISI scores are derived from the integrated embedding graph, while physical graphs are computed per sample. The final CLISIS aggregates scores across all samples.

GCS. The Graph Connectivity Similarity (GCS) is an unsupervised metric to compare the overlap between the graph connectivity of cells in the embedding feature space of a model with that in physical space. The GCS quantifies how well local spatial organization is preserved, and it ranges between 0 and 1, with 1 indicating perfect graph connectivity similarity and 0 indicating no graph connectivity similarity. We construct k-nearest neighbor graphs based on the physical and embedding spaces and measure dissimilarity using the Frobenius norm of their adjacency matrices. This value is normalized by the minimum graph connectivity overlap and scaled. In integration scenarios, GCS is calculated per sample using sample-specific graphs, and the mean is reported.

NASW. We define the Niche Average Silhouette Width (NASW) to measure the separability of clusters identified via clustering of the embedding space of a model. We construct a k-NN graph based on the embedding space, perform clustering across resolutions from 0.1 to 1.0 at 0.45 increments, and report the mean ASW across clusters.

CNMI. We compute the Cell Type Normalized Mutual Info (CNMI) using scIB[35].

BLISI and PCR. We compute the Batch Local Inverse Simpson's Index (BLISI) and Principal Component Regression score (PCR) from scIB[35].

Overall score. We compute an overall score through balanced aggregation of category scores. To obtain category scores for spatial consistency (s_s), niche coherence (s_n), and batch correction (s_b), we aggregate individual metrics pertaining to each category with equal weighting. Prior to aggregation, individual metrics are min-max scaled across all runs to ensure uniform contribution to the category scores. In single-sample scenarios, the overall score is calculated as: $s_o = \frac{1}{2}s_s + \frac{1}{2}s_n$. In integration scenarios, the overall score is calculated as: $s_o = \frac{1}{3}s_s + \frac{1}{3}s_n + \frac{1}{3}s_b$ with $s_s = \frac{1}{4}\text{CAS}^* + \frac{1}{4}\text{MLAMI}^* + \frac{1}{4}\text{CLISIS}^* + \frac{1}{4}\text{GCS}^*$, $s_n = \frac{1}{2}\text{NASW}^* + \frac{1}{2}\text{CNMI}^*$, and $s_b = \frac{1}{2}\text{BLISI}^* + \frac{1}{2}\text{PCR}^*$, where "*" indicates that metrics have been min-max scaled.

References

1. Lohoff, T. *et al.* Integration of spatial and single-cell transcriptomic data elucidates mouse organogenesis. *Nat. Biotechnol.* **40**, 74–85 (2022).
2. Moreno-Bravo, J. A. *et al.* Role of Shh in the development of molecularly characterized tegmental nuclei in mouse rhombomere 1. *Brain Struct. Funct.* **219**, 777–792 (2014).
3. Fausett, S. R., Brunet, L. J. & Klingensmith, J. BMP antagonism by Noggin is required in presumptive notochord cells for mammalian foregut morphogenesis. *Dev. Biol.* **391**, 111–124 (2014).
4. Wang, Q. *et al.* The Allen Mouse Brain Common Coordinate Framework: A 3D Reference Atlas. *Cell* **181**, 936–953.e20 (2020).
5. Singhal, V. *et al.* BANKSY unifies cell typing and tissue domain segmentation for scalable spatial omics data analysis. *Nat. Genet.* (2024) doi:10.1038/s41588-024-01664-3.
6. Long, Y. *et al.* Spatially informed clustering, integration, and deconvolution of spatial transcriptomics with GraphST. *Nat. Commun.* **14**, 1155 (2023).
7. Varrone, M., Tavernari, D., Santamaria-Martínez, A., Walsh, L. A. & Ciriello, G. CellCharter reveals spatial cell niches associated with tissue remodeling and cell plasticity. *Nat. Genet.* **56**, 74–84 (2024).
8. Li, R. & Yang, X. De novo reconstruction of cell interaction landscapes from single-cell spatial transcriptome data with DeepLinc. *Genome Biol.* **23**, 124 (2022).
9. Lein, E. S. *et al.* Genome-wide atlas of gene expression in the adult mouse brain. *Nature* **445**, 168–176 (2007).
10. Stickels, R. R. *et al.* Highly sensitive spatial transcriptomics at near-cellular resolution with Slide-seqV2. *Nat. Biotechnol.* **39**, 313–319 (2021).
11. Shi, H. *et al.* Spatial atlas of the mouse central nervous system at molecular resolution. *Nature* **622**, 552–561 (2023).
12. Xu, Z. *et al.* STOmicsDB: a database of spatial transcriptomic data. *bioRxiv* (2022).
13. Zhang, X., Wang, X., Shivashankar, G. V. & Uhler, C. Graph-based autoencoder integrates spatial transcriptomics with chromatin images and identifies joint biomarkers for Alzheimer's disease. *Nat. Commun.* **13**, 7480 (2022).
14. He, S. *et al.* High-plex imaging of RNA and proteins at subcellular resolution in fixed tissue by spatial molecular imaging. *Nat. Biotechnol.* **40**, 1794–1806 (2022).
15. Janesick, A. *et al.* High resolution mapping of the breast cancer tumor microenvironment using integrated single cell, spatial and in situ analysis of FFPE tissue. *bioRxiv* 2022.10.06.510405 (2022) doi:10.1101/2022.10.06.510405.
16. Morse, C. *et al.* Proliferating SPP1/MERTK-expressing macrophages in idiopathic pulmonary fibrosis. *Eur. Respir. J.* **54**, (2019).
17. Hoeft, K. *et al.* Platelet-instructed SPP1+ macrophages drive myofibroblast activation in fibrosis in a CXCL4-dependent manner. *Cell Rep.* **42**, 112131 (2023).
18. Ayaub, E. A. *et al.* Single Cell RNA-seq and Mass Cytometry Reveals a Novel and a Targetable Population of Macrophages in Idiopathic Pulmonary Fibrosis. *bioRxiv* 2021.01.04.425268 (2021) doi:10.1101/2021.01.04.425268.
19. Mayr, C. H. *et al.* Spatial transcriptomic characterization of pathologic niches in IPF. *Sci Adv* **10**, eadl5473 (2024).
20. Ridray, S. *et al.* Coexpression of dopamine D1 and D3 receptors in islands of Calleja and shell of nucleus accumbens of the rat: opposite and synergistic functional interactions. *Eur. J. Neurosci.* **10**, 1676–1686 (1998).
21. Zhang, D. *et al.* Spatial epigenome–transcriptome co-profiling of mammalian tissues. *Nature* 1–10 (2023).
22. Fey, M. & Lenssen, J. E. Fast Graph Representation Learning with PyTorch Geometric. *arXiv [cs.LG]* (2019).
23. Virshup, I. *et al.* The scverse project provides a computational ecosystem for single-cell omics data analysis. *Nat. Biotechnol.* **41**, 604–606 (2023).
24. Lotfollahi, M. *et al.* Mapping single-cell data to reference atlases by transfer learning. *Nat. Biotechnol.* (2021) doi:10.1038/s41587-021-01001-7.
25. Ramalho-Santos, M., Melton, D. A. & McMahon, A. P. Hedgehog signals regulate multiple aspects of gastrointestinal development. *Development* **127**, 2763–2772 (2000).
26. Zhang, X. M., Ramalho-Santos, M. & McMahon, A. P. Smoothened mutants reveal redundant roles for Shh and Ihh signaling including regulation of L/R asymmetry by the mouse node. *Cell* **105**, 781–792 (2001).
27. van den Brink, G. R. Hedgehog signaling in development and homeostasis of the gastrointestinal tract. *Physiol. Rev.* **87**, 1343–1375 (2007).

28. Reigstad, L. J., Varhaug, J. E. & Lillehaug, J. R. Structural and functional specificities of PDGF-C and PDGF-D, the novel members of the platelet-derived growth factors family. *FEBS J.* **272**, 5723–5741 (2005).

29. Mecklenburg, N. *et al.* Growth and differentiation factor 10 (Gdf10) is involved in Bergmann glial cell development under Shh regulation. *Glia* **62**, 1713–1723 (2014).

30. Traiffort, E., Angot, E. & Ruat, M. Sonic Hedgehog signaling in the mammalian brain. *J. Neurochem.* **113**, 576–590 (2010).

31. Cohen, M. *et al.* Ptch1 and Gli regulate Shh signalling dynamics via multiple mechanisms. *Nat. Commun.* **6**, 6709 (2015).

32. Park, E. *et al.* EphA/ephrin-A signaling is critically involved in region-specific apoptosis during early brain development. *Cell Death Differ.* **20**, 169–180 (2013).

33. Lopez, R., Regier, J., Cole, M. B., Jordan, M. I. & Yosef, N. Deep generative modeling for single-cell transcriptomics. *Nat. Methods* **15**, 1053–1058 (2018).

34. Lotfollahi, M. *et al.* Biologically informed deep learning to query gene programs in single-cell atlases. *Nat. Cell Biol.* **25**, 337–350 (2023).

35. Luecken, M. D. *et al.* Benchmarking atlas-level data integration in single-cell genomics. *Nat. Methods* **19**, 41–50 (2022).

36. Korsunsky, I. *et al.* Fast, sensitive and accurate integration of single-cell data with Harmony. *Nat. Methods* **16**, 1289–1296 (2019).

37. Heidari, E. *et al.* Supervised spatial inference of dissociated single-cell data with SageNet. *bioRxiv* 2022.04.14.488419 (2022) doi:10.1101/2022.04.14.488419.

38. Yuan, Z. *et al.* Benchmarking spatial clustering methods with spatially resolved transcriptomics data. *Nat. Methods* **21**, 712–722 (2024).

39. Badia-I-Mompel, P. *et al.* decoupleR: ensemble of computational methods to infer biological activities from omics data. *Bioinform Adv* **2**, vbac016 (2022).

40. Dimitrov, D. *et al.* Comparison of methods and resources for cell-cell communication inference from single-cell RNA-Seq data. *Nat. Commun.* **13**, 3224 (2022).

41. Hsieh, Y.-C. & Puche, A. C. Development of the Islands of Calleja. *Brain Res.* **1490**, 52–60 (2013).

42. Rodrigo, J. *et al.* Physiology and pathophysiology of nitric oxide in the nervous system, with special mention of the islands of Calleja and the circunventricular organs. *Histol. Histopathol.* **17**, 973–1003 (2002).

43. Eguren, M., Manchado, E. & Malumbres, M. Non-mitotic functions of the Anaphase-Promoting Complex. *Semin. Cell Dev. Biol.* **22**, 572–578 (2011).

44. Rodriguez-Rodriguez, P., Almeida, A. & Bolaños, J. P. Brain energy metabolism in glutamate-receptor activation and excitotoxicity: role for APC/C-Cdh1 in the balance glycolysis/pentose phosphate pathway. *Neurochem. Int.* **62**, 750–756 (2013).

45. Wang, J. *et al.* POU2F1 Promotes Cell Viability and Tumor Growth in Gastric Cancer through Transcriptional Activation of lncRNA TTC3-AS1. *J. Oncol.* **2021**, 5570088 (2021).

46. Mergenthaler, P., Lindauer, U., Dienel, G. A. & Meisel, A. Sugar for the brain: the role of glucose in physiological and pathological brain function. *Trends Neurosci.* **36**, 587–597 (2013).

47. Fieblinger, T. Striatal Control of Movement: A Role for New Neuronal (Sub-) Populations? *Front. Hum. Neurosci.* **15**, 697284 (2021).

48. Laitman, B. M. *et al.* The Transcriptional Activator Krüppel-like Factor-6 Is Required for CNS Myelination. *PLoS Biol.* **14**, e1002467 (2016).

49. Mela, A. & Goldman, J. E. The tetraspanin KAI1/CD82 is expressed by late-lineage oligodendrocyte precursors and may function to restrict precursor migration and promote oligodendrocyte differentiation and myelination. *J. Neurosci.* **29**, 11172–11181 (2009).

50. Shao, Z. *et al.* LINGO-1 Regulates Oligodendrocyte Differentiation through the Cytoplasmic Gelsolin Signaling Pathway. *J. Neurosci.* **37**, 3127–3137 (2017).

51. Baker, S. A., Gajera, C. R., Wawro, A. M., Corces, M. R. & Montine, T. J. GATM and GAMT synthesize creatine locally throughout the mammalian body and within oligodendrocytes of the brain. *Brain Res.* **1770**, 147627 (2021).

52. Takase, H. *et al.* Transcriptome Profiling of Mouse Corpus Callosum After Cerebral Hypoperfusion. *Front Cell Dev Biol* **9**, 685261 (2021).

53. Samtani, G. *et al.* Brain region dependent molecular signatures and myelin repair following chronic demyelination. *Front. Cell. Neurosci.* **17**, 1169786 (2023).

54. de Monasterio-Schrader, P. *et al.* Uncoupling of neuroinflammation from axonal degeneration in mice lacking the myelin protein tetraspanin-2. *Glia* **61**, 1832–1847 (2013).

55. Gaudet, P., Livstone, M. S., Lewis, S. E. & Thomas, P. D. Phylogenetic-based propagation of functional annotations within the Gene Ontology consortium. *Brief. Bioinform.* **12**, 449–462 (2011).

56. Wolf, F. A., Angerer, P. & Theis, F. J. SCANPY: large-scale single-cell gene expression data analysis.
