# Supplementary material for: Quantitative characterization of cell niches in spatially resolved omics data
Source: Nat Genet. 2025 Mar 18;57(4):897–909. doi: 10.1038/s41588-025-02120-6 (PMC11985353; doi:10.1038/s41588-025-02120-6)
Supplement: Supplementary file 2 — Reporting Summary [file 41588_2025_2120_MOESM2_ESM.pdf]

nature portfolio

Corresponding author(s): Prof. Dr. Carlos Talavera-Loópez and Dr. Mohammad Lotfollahi

Last updated by author(s): 08.01.2025

Reporting Summary

Nature Portfolio wishes to improve the reproducibility of the work that we publish. This form provides structure for consistency and transparency in reporting. For further information on Nature Portfolio policies, see our Editorial Policies and the Editorial Policy Checklist.

Statistics

For all statistical analyses, confirm that the following items are present in the figure legend, table legend, main text, or Methods section.

n/a	Confirmed	
☐	☒	The exact sample size (*n*) for each experimental group/condition, given as a discrete number and unit of measurement
☐	☒	A statement on whether measurements were taken from distinct samples or whether the same sample was measured repeatedly
☐	☒	The statistical test(s) used AND whether they are one- or two-sided<br>*Only common tests should be described solely by name; describe more complex techniques in the Methods section.*
☐	☒	A description of all covariates tested
☐	☒	A description of any assumptions or corrections, such as tests of normality and adjustment for multiple comparisons
☐	☒	A full description of the statistical parameters including central tendency (e.g. means) or other basic estimates (e.g. regression coefficient) AND variation (e.g. standard deviation) or associated estimates of uncertainty (e.g. confidence intervals)
☐	☒	For null hypothesis testing, the test statistic (e.g. *F*, *t*, *r*) with confidence intervals, effect sizes, degrees of freedom and *P* value noted<br>*Give P values as exact values whenever suitable.*
☐	☒	For Bayesian analysis, information on the choice of priors and Markov chain Monte Carlo settings
☒	☐	For hierarchical and complex designs, identification of the appropriate level for tests and full reporting of outcomes
☒	☐	Estimates of effect sizes (e.g. Cohen's *d*, Pearson's *r*), indicating how they were calculated

Our web collection on statistics for biologists contains articles on many of the points above.

Software and code

Policy information about availability of computer code

Data collection	To collect default ligand-receptor and transcriptional regulation programs, we used omnipath (version 1.0.8) and decoupler (version 1.8.0) with Python version 3.9.19, respectively. Default metabolite-sensor programs were retrieved from https://github.com/zhengrongbin/MEBOCOST (on 18.05.2023). Default combined interaction programs were constructed using NicheNet's regulatory potential matrix, retrieved from https://zenodo.org/record/7074291. No spatial omics data was collected as part of this study (all data was previously published). R source data files were collected and converted to AnnData objects using R (version 4.4.0) with the following libraries: Bioconductor (version 3.20), rhdf5 (version 2.50.1), scRNAseq (version 2.20.0), Seurat (version 5.1.0), SeuratData (version 0.2.2.9001), SeuratDisk (version 0.0.0.9021), and zellkonverter (version 1.16.0). Package versions of our R and Python environments are also available at https://github.com/Lotfollahi-lab/nichecompass-reproducibility.
Data analysis	All experiments were conducted with Python version 3.9.19 and R version 4.4.0 unless otherwise specified. All experiments were performed with NicheCompass version 0.1.2 (except ablation study experiments which were performed with NicheCompass version 0.2.0). Other major Python software that we used for data analysis included: altair (5.5.0), anndata (version 0.10.8), GraphST (version 1.1.1), matplotlib (version 3.8.4), liana (version 1.3.0), networkx (version 3.2.1), numpy (1.26.4), pandas (2.2.3), plotly (version 5.24.1), plottable (version 0.1.5), pyensembl (2.3.13), pynrrd (version 1.0.0), pywaffle (version 0.0.8), scanpy (version 1.9.8), scarches (version 0.5.9), scib-metrics (version 0.5.1), scikit-learn (version 1.6.1), scipy (1.12.0), seaborn (version 0.13.2), skimage (version 0.24.0), squidpy (version 1.6.1), tiledb (version 0.20.0), tiledbsoma (version 0.1.22), torch (version 2.0.0), torch_geometric (version 2.5.3). We copied the BANKSY source code from https://github.com/prabhakarlab/Banksy (12.07.2024). We copied the STACI source code from https://github.com/uhlerlab/STACI/blob/master (23.11.2023). The data simulation was performed with the following R libraries: srtSIM (version 0.99.6), readr (version 2.1.5), data.table (version 1.16.4), dplyr (version 1.1.4), SingleCellExperiment (version 1.28.1), and zellkonverter (version 1.16.0). Package versions of our R and Python environments are also available at https://github.com/Lotfollahi-lab/nichecompass-reproducibility. DeepLinc was run with Python version 3.7.0 and its dependencies are available in a separate yaml file at https://github.com/Lotfollahi-lab/nichecompass-reproducibility.

CellCharter (version 0.3.2) and scvi -tools (version 0.20.3) were run with Python version 3.10.0 and dependencies that are available in a separate yaml file at https://github.com/Lotfollahi-lab/nichecompass-reproducibility. STalign (version 1.0.1) was run with Python version 3.9.19 and its dependencies are available in a separate yaml file at https://github.com/Lotfollahi-lab/nichecompass-reproducibility.

For manuscripts utilizing custom algorithms or software that are central to the research but not yet described in published literature, software must be made available to editors and reviewers. We strongly encourage code deposition in a community repository (e.g. GitHub). See the Nature Portfolio guidelines for submitting code & software for further information.

Data

Policy information about availability of data

All manuscripts must include a data availability statement. This statement should provide the following information, where applicable:

- Accession codes, unique identifiers, or web links for publicly available datasets
- A description of any restrictions on data availability
- For clinical datasets or third party data, please ensure that the statement adheres to our policy

All datasets used in this study were previously published. Processed versions are available as AnnData objects for download as outlined at https://github.com/Lotfollahi-lab/nichecompass-reproducibility. The seqFISH mouse organogenesis dataset was sourced from https://marionilab.cruk.cam.ac.uk/SpatialMouseAtlas/. The SlideSeqV2 dataset was obtained via squidpy.datasets.slideseqv2(). The MERFISH mouse liver dataset was retrieved from https://info.vizgen.com/mouse-liver-access (animal 1, replicate 1). The nanoString CosMx NSCLC dataset was collected from https://nanostring.com/products/cosmx-spatial-molecular-imager/ffpe-dataset/nsclc-ffpe-dataset/. The Xenium human breast cancer dataset was downloaded from https://www.10xgenomics.com/products/xenium-in-situ/preview-dataset-human-breast. The STARmap PLUS mouse CNS dataset was obtained from https://zenodo.org/records/8327576. The MERFISH whole mouse brain dataset was retrieved from https://cellxgene.cziscience.com/collections/0cca8620-8dee-45d0-aef5-23f032a5cf09. The Spatial ATAC-RNA seq mouse brain dataset (postnatal day 22) was collected from https://www.ncbi.nlm.nih.gov/geo/query/acc.cgi?acc=GSE205055 (gene expression counts and spatial coordinates) and https://brain-spatial-omics.cells.ucsc.edu/ (peak counts and cell type labels). Lastly, the Stereo-seq mouse embryo dataset was downloaded from http://sdmbench.drai.cn/ (Data ID 13).

Research involving human participants, their data, or biological material

Policy information about studies with human participants or human data. See also policy information about sex, gender (identity/presentation), and sexual orientation and race, ethnicity and racism.

Reporting on sex and gender	No human data were collected as part of the study.
Reporting on race, ethnicity, or other socially relevant groupings	No human data were collected as part of the study.
Population characteristics	No human data were collected as part of the study.
Recruitment	No human data were collected as part of the study.
Ethics oversight	No human data were collected as part of the study.

Note that full information on the approval of the study protocol must also be provided in the manuscript.

Field-specific reporting

Please select the one below that is the best fit for your research. If you are not sure, read the appropriate sections before making your selection.

☒ Life sciences ☐ Behavioural & social sciences ☐ Ecological, evolutionary & environmental sciences

For a reference copy of the document with all sections, see nature.com/documents/nr-reporting-summary-flat.pdf

Life sciences study design

All studies must disclose on these points even when the disclosure is negative.

Sample size	No statistical method was used to predetermine sample size, and no data were excluded from the analyses unless explicitly stated. The seqFISH mouse organogenesis dataset included 57,536 cells across six sagittal tissue sections from three 8-12 somite stage mouse embryos: 19,451 (embryo 1), 14,891 (embryo 2), and 23,194 (embryo 3). The SlideSeqV2 mouse hippocampus dataset included 41,786 observations at near-cellular resolution. The MERFISH mouse liver dataset included 367,335 cells. The nanoString CosMx human NSCLC dataset included 800,327 cells across 8 tissue sections from 5 donors. Cell counts per section are: 93,206 cells (donor 1, replicate 1), 93,206 cells (donor 1, replicate 2), 91,691 cells (donor 1, replicate 3), 91,691 cells (donor 2), 77,391 cells (donor 3, replicate 1), 115,676 (donor 3, replicate 2), 66,489 cells (donor 4) and 76,536 cells (donor 5). The Xenium human breast cancer dataset included 282,363 cells across two replicates (replicate 1: 164,000, replicate 2: 118,363). The STARmap PLUS mouse CNS dataset included 1,091,527 million cells. For ablation studies, only the first sagittal tissue section was used (91,246 cells). The MERFISH whole mouse brain dataset included ~8.4 million cells across 239 sections from four animals. The Spatial ATAC-RNA seq mouse brain dataset included 9,215 spot-level observations. The Stereo-seq mouse embryo dataset included 5,913 spot-level observations.

Data exclusions	In the seqFISH mouse organogenesis dataset, we filtered cells annotated as low quality by the original authors. In the nanoString CosMx human NSCLC dataset, we filtered cells with < 50 counts, which were assessed to be low quality cells after QC.
Replication	For method benchmarking, we performed n = 8 training runs for each method. For ablation studies, we performed n = 8 training runs for each model configuration. For data analysis, we trained a single NicheCompass model per dataset unless otherwise specified. In the seqFISH mouse organogenesis dataset, we evaluated robustness and reproducibility of analysis results by repeating training runs across n = 3 seeds and n = 4 neighborhood graphs, confirming that results are robust and reproducible.
Randomization	To generate simulated data, we randomly sampled (1) niches to upregulate programs, (2) increment parameters with which programs were upregulated, (3) source and target cell types of programs, and (4) prior programs and (5) program member genes to be upregulated. Different random seeds were used for different runs during benchmarking.
Blinding	Blinding was not applicable to this study because no sample group allocation was performed.

Reporting for specific materials, systems and methods

We require information from authors about some types of materials, experimental systems and methods used in many studies. Here, indicate whether each material, system or method listed is relevant to your study. If you are not sure if a list item applies to your research, read the appropriate section before selecting a response.

Materials & experimental systems

n/a	Involved in the study	
☒	☐	Antibodies
☒	☐	Eukaryotic cell lines
☒	☐	Palaeontology and archaeology
☒	☐	Animals and other organisms
☒	☐	Clinical data
☒	☐	Dual use research of concern
☒	☐	Plants

Methods

n/a	Involved in the study	
☒	☐	ChIP-seq
☒	☐	Flow cytometry
☒	☐	MRI-based neuroimaging

Plants

Seed stocks	No plant data were collected or analyzed as part of this study.
Novel plant genotypes	No plant data were collected or analyzed as part of this study.
Authentication	No plant data were collected or analyzed as part of this study.
